# The C-terminal domain of the antiamyloid chaperone DNAJB6 binds to amyloid-β peptide fibrils and inhibits secondary nucleation

**DOI:** 10.1016/j.jbc.2023.105317

**Published:** 2023-10-04

**Authors:** Nicklas Österlund, Rebecca Frankel, Andreas Carlsson, Dev Thacker, Maja Karlsson, Vanessa Matus, Astrid Gräslund, Cecilia Emanuelsson, Sara Linse

**Affiliations:** 1 Department of Biochemistry and Biophysics, Stockholm University, Stockholm, Sweden; 2 Division of Biochemistry and Structural Biology, Department of Chemistry, Lund University, Lund, Sweden

**Keywords:** amyloid-beta, chaperone DNAJ, neurodegeneration, protein aggregation, self-assembly, protein engineering

## Abstract

The DNAJB6 chaperone inhibits fibril formation of aggregation-prone client peptides through interaction with aggregated and oligomeric forms of the amyloid peptides. Here, we studied the role of its C-terminal domain (CTD) using constructs comprising either the entire CTD or the first two or all four of the CTD β-strands grafted onto a scaffold protein. Each construct was expressed as WT and as a variant with alanines replacing five highly conserved and functionally important serine and threonine residues in the first β-strand. We investigated the stability, oligomerization, antiamyloid activity, and affinity for amyloid-β (Aβ42) species using optical spectroscopy, native mass spectrometry, chemical crosslinking, and surface plasmon resonance technology. While DNAJB6 forms large and polydisperse oligomers, CTD was found to form only monomers, dimers, and tetramers of low affinity. Kinetic analyses showed a shift in inhibition mechanism. Whereas full-length DNAJB6 activity is dependent on the serine and threonine residues and efficiently inhibits primary and secondary nucleation, all CTD constructs inhibit secondary nucleation only, independently of the serine and threonine residues, although their dimerization and thermal stabilities are reduced by alanine substitution. While the full-length DNAJB6 inhibition of primary nucleation is related to its propensity to form coaggregates with Aβ, the CTD constructs instead bind to Aβ42 fibrils, which affects the nucleation events at the fibril surface. The retardation of secondary nucleation by DNAJB6 can thus be ascribed to the first two β-strands of its CTD, whereas the inhibition of primary nucleation is dependent on the entire protein or regions outside the CTD.

DnaJ/Hsp40 proteins are important ATP-independent chaperones that bind to specific client proteins and deliver them to the Hsp70 machinery (1). The DnaJ family includes 45 different homologs in human, divided into three subfamilies (A, B, and C), of which the B subfamily has been shown to be linked to suppression of protein aggregation (2). The B subfamily can be further divided into a classical (DNAJB1, and related) and a nonclassical (DNAJB6, DNAJB8, and related) cluster. DNAJB1-like chaperones participate in the eukaryotic protein disaggregation system that increases the rate of amyloid fibril dissociation (3, 4). DNAJB6 has been found to massively retard the formation of amyloid fibrils and to affect the equilibrium solubility of amyloid peptides (5, 6). Among the proteins for which amyloid formation is reported to be suppressed by DNAJB6 *in vivo* and *in vitro* are poly-Q peptides from Huntington’s disease (7, 8, 9, 10), α-synuclein from Parkinson’s disease (11, 12), and amyloid-β (Aβ) peptides Aβ40 (13) and Aβ42 (5, 6) from Alzheimer’s disease. The Aβ42 peptide, the client studied in this current work, aggregates *via* a double nucleation mechanism (14). Primary nucleation of monomers in solution is a very slow event, whereas the energy barrier is significantly reduced for fibril-dependent secondary nucleation of monomers, leading to a much faster nucleation of amyloid (14, 15). Elongation of existing fibrils by monomer addition is associated with the lowest energy barrier (15). The effect of DNAJB6 on Aβ42 aggregation is due to not only strong suppression of primary nucleation but also suppression of secondary nucleation (5). The presence of the chaperone also shifts the equilibrium toward higher solubility of Aβ42 (6). Aβ aggregation is affected at remarkably low substoichiometric molar ratios of chaperone to client peptide, and the interaction is believed to be with oligomeric rather than monomeric or fibrillar forms of Aβ (5, 13). The exact mechanism and driving forces behind chaperone action and chaperone–client interactions, however, remain largely unknown.

DNAJB6 contains two globular domains, the J-domain (JD) and the C-terminal domain (CTD), connected by a relatively unstructured linker domain rich in serine (S), threonine (T), glycine, (G), and phenylalanine (F) residues, as seen in an AlphaFold2 prediction (Fig. 1*A*). The JD is highly conserved, is found in all DnaJ proteins, and interacts with Hsp70 *via* a conserved HPD motif (16, 17). J protein CTDs have considerably more structural diversity but have been found to be important for binding of client proteins (16, 17) and is in DNAJB proteins known to be critical for their antiaggregation function *in vivo* (2). DNAJB6-like J-proteins have one CTD that consists of a single β-sheet. DNAJB6 and its closest homologs also contain a characteristic stretch of highly conserved S/T residues in and just N-terminal to β-strand 1 (β1) of the CTD (Fig. 1*B*). Substitution of these S/T residues with alanines reduces the effects of DNAJB6 on both amyloid nucleation and Aβ solubility (6). A previously published dimer model of DNAJB6 suggests that the two β1 strands in the CTDs are solvent accessible in the dimer and in an antiparallel arrangement at the monomer–monomer interface (18) (Fig. 1*C*). Crosslinking mass spectrometry (MS) detects lysine-specific chemical crosslinks between Aβ42 and four lysine residues in the CTD (Fig. 1*C*, *purple spheres*) (18). No crosslinks are detected between Aβ42 and the JD, whereas the unstructured linker in DNAJB6 does not contain any lysines and is thus unavailable for crosslinking (18). This indicates that the CTD may be important for chaperone–client interactions in DNAJB6. Peptide docking in AlphaFold2 does indeed suggest a complex where Aβ interacts preferentially with the S/T-rich β1 in the CTD (Fig. 1*D*) (19).

**Figure 1:**
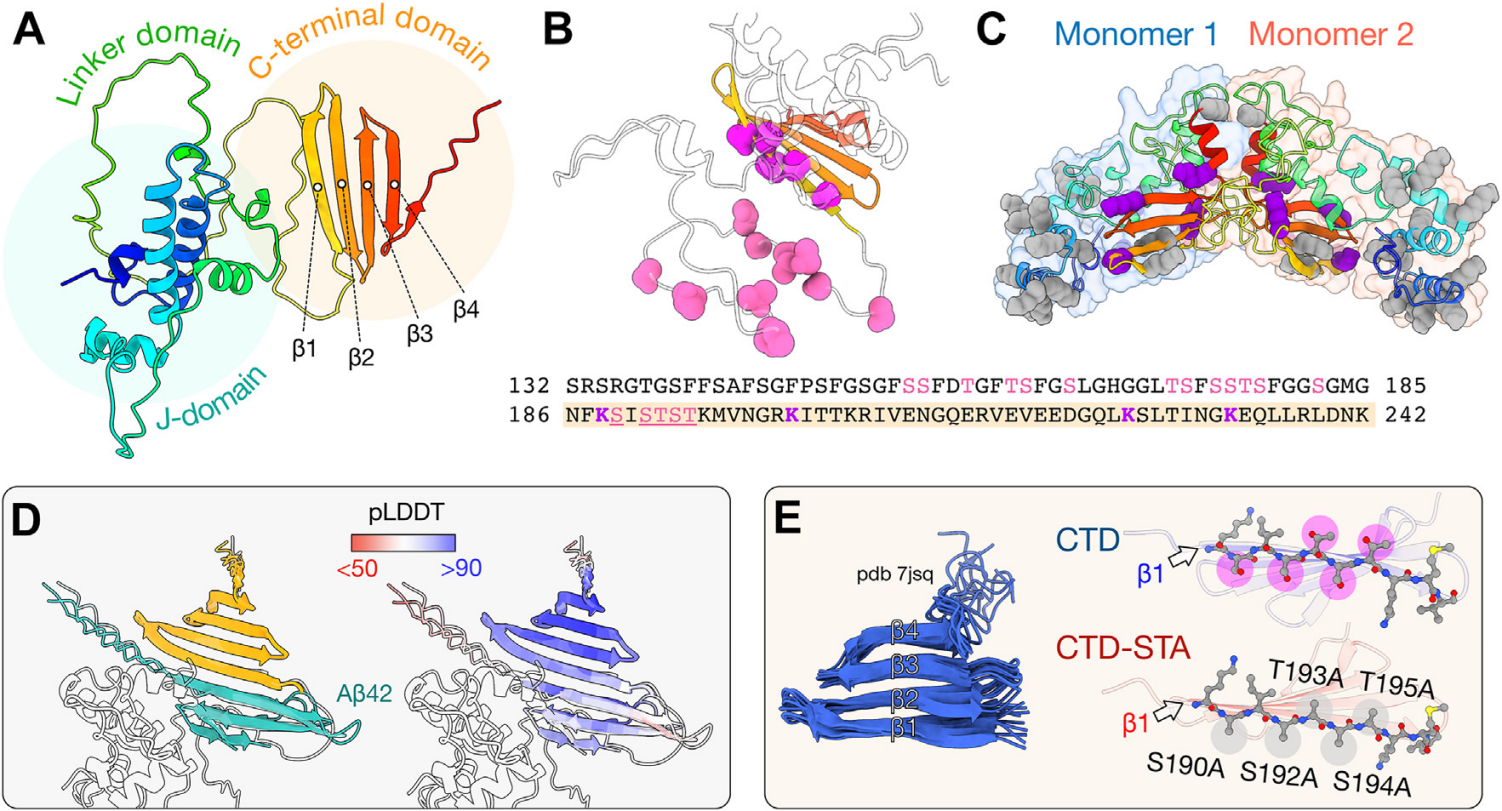
**Structural outline DNAJB6 and its C-terminal domain (CTD).***A*, structural model of DNAJB6b as predicted by AlphaFold2. The protein is colored according to sequence position from *blue* (N-terminal end) to *red* (C-terminal end). *B*, residues 132 to 242 in the AlphaFold2 model, including the linker and CTD with the S/T residues that have been found to be of importance for the antiamyloid effect of full-length DNAJB6, highlighted as *spheres* in *hot pink* (5 residues in in β-strand 1) and *pink* (ten residues in the linker). *C*, previously published DNAJB6 dimer model as predicted by HADDOCK docking with two experimental restrains (18), in which two CTDs are positioned at the dimer interface. Lysine residues are shown as *spheres*, with lysines found to crosslink with Aβ42 colored *purple* (18). *D*, peptide docking of Aβ42 to DNAJB6 residues 132 to 242 in AlphaFold2, showing the three top ranked models overlaid. *Left*, colored according to chain, CTD in *orange*, Aβ42 in *cyan*. *Right*, colored according to pLDDT score (*red* to *blue*). *E*, NMR structure of the independently folded CTD (Protein Data Bank code: 7JSQ (22)), which is studied here. Residues in β1 are highlighted as *sticks* for the CTD construct and the S/T-A-substituted variant (CTD STA), which is also studied.

DNAJB6 has a high tendency to form highly polydisperse oligomers, in contrast to DNAJB1 that functions as a dimer (20, 21). The polydispersity of DNAJB6 is seen as a broad elution in size-exclusion chromatography (SEC) and a lack of discrete bands on native electrophoresis gels (7). Electron microscopy reveals a loose oligomeric structure full of voids and only small interaction surfaces between the proteins in the oligomers (18). The self-assembly of DNAJB6 into oligomers is partly dependent on the CTD and partly on the functionally important S/T-rich region in the unstructured linker domain, as deletion of 20 or 55 amino acid residues (Δ132–183) before β1 of CTD results in a reduction of oligomers and an increase in monomer concentration (6, 22). NMR data point toward an important role of the five S/T residues in β1 for strand twisting, intermolecular strand–strand interactions, and chaperone self-affinity. The NMR data further imply that the T193A point mutation decreases the CTD dimerization/oligomerization propensity and affects the local dynamics of β1 (23, 24). *In vivo*, the T193A mutation is associated with altered α-synuclein homeostasis and Parkinson’s disease (25), indicating a possible correlation between DNAJB6 oligomerization and chaperone activity.

This study was motivated by the potent inhibition by DNAJB6 of both primary and secondary nucleation of Aβ42, the more aggregation-prone of the Aβ alloforms. We ask whether the effects of full-length DNAJB6 on Aβ42 fibril formation kinetics and oligomer formation can be mimicked by the CTD, the putative client-binding domain. The CTD was earlier found to be an independent folding unit (22, 23) and is here studied *in vitro* in isolation and together with Aβ42. *In vitro* studies offer tighter control over the composition of systems, and previous studies have found that the same mechanistic steps describe aggregation in buffer and cerebrospinal fluid (CSF) (26), and that inhibitors act on the same steps in both environments (27). One construct with the DNAJB6 WT sequence (residues 186–242 plus a starting Met), referred to as CTD, and one variant with five S/T to A substitutions in β1 (S190A + S192A + T193A + S194A + T195A), referred to as CTD STA, are studied (Fig. 1*E*). This STA variant was designed based on the previously observed decrease in antiamyloid activity of full-length DNAJB6 with these substitutions (6) to study whether this effect is replicated by the isolated CTD. Shorter segments comprising β1–β2 or β1–β4 of CTD grafted onto S100G, referred to as S100G-CTDβ1–2 and S100G-CTDβ1–4 (Fig. S1), are studied to further pinpoint the interactions with Aβ42. The effect of each construct on Aβ42 aggregation is monitored using thioflavin T (ThT) fluorescence, the interaction with Aβ42 fibrils using surface resonance technology, and the formation of small coaggregates with Aβ42 by native MS. The stability toward thermal denaturation is studied using CD spectroscopy and native MS. The Ca^2+^-binding affinity and cooperativity for S100G-grafted constructs are studied using a competitive spectroscopic assay to monitor any perturbations of the host scaffold.

## Results

### The stability toward unfolding of the CTD

**Figure 2:**
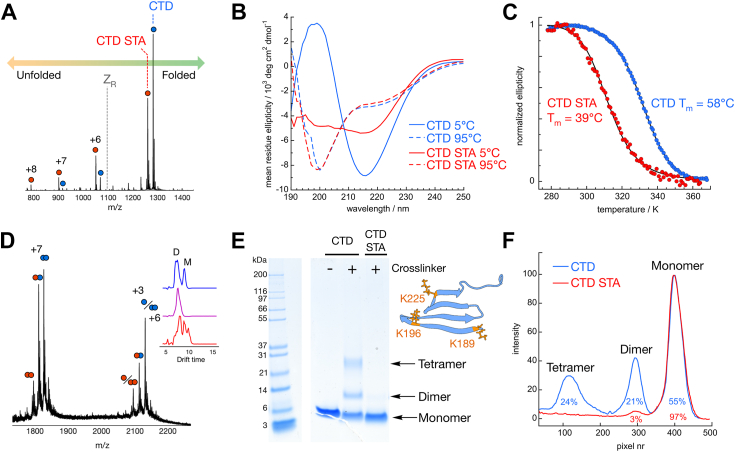
**Structural characterization of C-terminal domain (CTD) constructs.***A*, native MS of an equimolar mixture of CTD and CTD STA as ionized from 200 mM ammonium acetate (pH 6.8). The charge state distribution is shifted toward higher charge states for CTD STA (*red*) compared with CTD (*blue*). The Rayleigh limit (z_R_) is indicated, which is the maximum theoretical charge that a spherically folded CTD protein could acquire during electrospray ionization. Charge states higher than z_R_ likely correspond to partially unfolded structural states. *B*, far-UV CD spectra of CTD (*blue*) and CTD STA (*red*) at 5 °C (*solid lines*), at 95 °C (*dashed lines*) in 20 mM sodium phosphate, pH 8.0. *C*, thermal denaturation of CTD and CTD STA as monitored by the CD signal at 216 nm. *D*, dimer regime of the mass spectrum for the mixture of CTD and CTD STA, indicating the presence of both homodimers and heterodimers, with CTD having a higher dimerization propensity compared with CTD STA. An insert of the ion mobility drift times for the overlapping n/z = 1/3, 2/6 peak is shown. *E*, SDS-PAGE analysis of CTD and CTD STA with (+) and without (−) crosslinking prior to analysis. The uncropped gel is shown in Fig. S12*A*. The residues involved in the detected crosslinks are shown in the structural model of CTD; for further details, see Table 1. *F*, ImageJ analysis of the lanes after crosslinking, with the intensity calculated as 250 Gy value, normalized from 0 to 100, and the percentage of each species deduced from peak integration indicated under the respective peak.

Far-UV CD spectroscopy reports on the average secondary structure content in a sample. CTD displays a far-UV CD spectrum typical of a β-sheet-rich protein in 20 mM sodium phosphate, pH 8.0, at 5 °C, with a minimum at 216 nm and a maximum at 198 nm (Fig. 2*B*, *blue trace*). CTD STA displays an altered spectrum with less negative ellipticity at 216 nm and no peak at 205 nm (Fig. 2*B*, *red trace*). Both proteins seem to denature completely at high temperature with spectra typical of random coil observed at 95 °C (Fig. 2*B*, *dashed lines*). The thermal denaturation data indicate that the temperature at the denaturation midpoint (*T*_m_) is 58 °C for CTD but only 39 °C for CTD STA (Fig. 2*C*). Neither protein refolds completely upon returning to 5 °C (Fig. S2), after which CTD STA is retained in a mostly unstructured state, whereas CTD is partially refolded to an intermediate state, with similar spectrum as the initial state of CTD STA.

The charge state distributions of ionized CTD and CTD STA were analyzed using electrospray MS. The proteins were ionized from 200 mM ammonium acetate, pH 6.8, at a source temperature of 25 °C. Electrospray MS was performed using very gentle ionization settings that are able to retain noncovalent interactions upon transfer to the gas phase (so-called “native” MS). CTD and CTD STA samples were mixed for native MS analysis, as the difference in mass between the constructs makes them easily separable in the *m/z* dimension. Analyzing both proteins in the same samples eliminate errors that could be due to variations in ionization efficiency between samples.

Both proteins are observed as mostly monomers after ionization in native MS (Fig. 2*A*), with narrow charge state distribution centered around +5. Charging in electrospray is related to solvent-accessible surface area, and narrow low-charged distributions consequently correspond to well-ordered and compact protein structures with small solvent-accessible surface areas. The ion mobility of the +5 ions confirms a collision cross section in agreement with the published NMR structure of CTD (Fig. S3). The Rayleigh charge (Z_R_) is the highest charge that a spherical droplet of a certain size can hold. Protein charge states higher than the Z_R_ of the protein cannot form from ionization of a compact folded structure and instead correspond to more extended structures (28, 29). Charge state analysis of the CTD proteins show charge states higher than +6, which are thus likely to represent such extended states for these proteins. The charge state of CTD STA is shifted toward higher charges, reaching all the way up to +8 (Fig. 2*A*). This indicates that the population of CTD STA is shifted toward more unstructured states compared with CTD, in agreement with its CD spectrum and its lower stability toward thermal denaturation (Fig. 2, *B* and *C*).

### Oligomerization propensity of the CTD

Although both protein constructs are seen as mostly monomeric, a small population of dimers could be observed using native MS (Fig. 2*C*). This dimeric population was larger for CTD compared with CTD STA, indicating that the S/T->A amino acid residue substitutions affect also the propensity to dimerize. Also, hetero-CTD/CTD STA dimers do seem to form. These heterodimers are, however, shifted toward higher charge states compared with CTD homodimers, indicating a shift toward slightly more extended structures. The results regarding shifts in charge state, extendedness, and dimerization propensity were also confirmed using nonmixed samples of CTD and CTD STA (Fig. S3). Oligomerization was further studied using chemical crosslinking of samples containing 10 μM of CTD or CTD STA in 20 mM sodium phosphate, 0.2 mM EDTA (pH 8.0), followed by SDS-PAGE and MS/MS analysis of the crosslinked peptides. This analysis reveals an increased oligomerization propensity of WT CTD compared with CTD STA. Both dimers and tetramers of CTD could be detected by SDS-PAGE (Fig. 2*E*) after crosslinking with the amine-reactive BS^3^ crosslinker, which crosslinks lysine residues within ca. 30 Å, corresponding to slightly more than the diameter of a CTD monomer, whereas CTD STA only displayed a weak band corresponding to the dimeric species and mainly a monomeric band. The oligomerization propensity and polydispersity of CTD is significantly lower than for full-length DNAJB6 (13).

Based on the integrated intensity of gel bands corresponding to monomers, dimers, and tetramers (Fig. 2*F*), and the total concentration of each construct (10 μM), the concentration of each species was calculated assuming equal staining per monomer in each species. The analysis further assumes that the crosslinking reaction is fast compared with monomer–dimer and dimer–tetramer exchange. If not, the estimates for *K*_*D*_ represent lower bounds of their values (upper bound of the affinity). We can thus estimate the dimer-to-monomer equilibrium dissociation constant (*K*_*D*_ = [monomer]^2^/[dimer]) to *K*_*D*_ ≥30 μM (=5.5^2^ μM/1.05) for CTD and *K*_*D*_ ≥600 μM (=9.7^2^ μM/0.15) for CTD STA and the tetramer-to-dimer equilibrium dissociation constant (*K*_*D*_ = [dimer]^2^/[tetramer]) to *K*_*D*_ ≥2 μM (=1.05^2^ μM/0.6) for CTD. LC–MS/MS analysis of the excised dimeric CTD band leads to identification of a crosslink between K189 and K189, thus crosslinking β-strand 1 in one monomer with β-strand 1 in the other monomer in the dimer. The K189–K189 crosslink was also observed in tetrameric CTD, as well as additional crosslinks within 30 Å distance, between K189 and K225 and between K196 and K225 in β-strand 4. The detected crosslinked peptides are presented in Table 1. A prediction of the dimeric CTD structure using AlphaFold2 suggests an interaction between β-strand 1 in each monomer in an antiparallel arrangement (Fig. S4, *A*–*C*). We also find that AlphaFold modeling predicts that dimerization is impeded by the S/T->A substitutions (Fig. S4, *D*–*F*).

**Table 1:** Detected crosslinks in bands corresponding to CTD dimer and CTD oligomer

Sample	Precursor mass	Charge	Score	Crosslinked peptides	Lys A^a^	Lys B^a^
CTD dimer	958.172	3+	20.1	KSISTSTKMVNGR + KSISTSTKM	K189	K189
CTD tetramer	958.172	3+	17.6	KSISTSTKMVNGR + KSISTSTKM	K189	K189
	683.956	5+	24.8	KSISTSTKMVNGR + VEVEEDGQLKSLTINGK	K189	K225
	658.337	6+	24.1	SISTSTKMVNGR + VEVEEDGQLKSLTINGK	K196	K225

a Lysine residue numbers refer to the number in full-length DNAJB6. Approved MS/MS spectra to validate the crosslinks are supplied in Fig. S5, *A*–*D*.

### Antiamyloid activity

**Figure 3:**
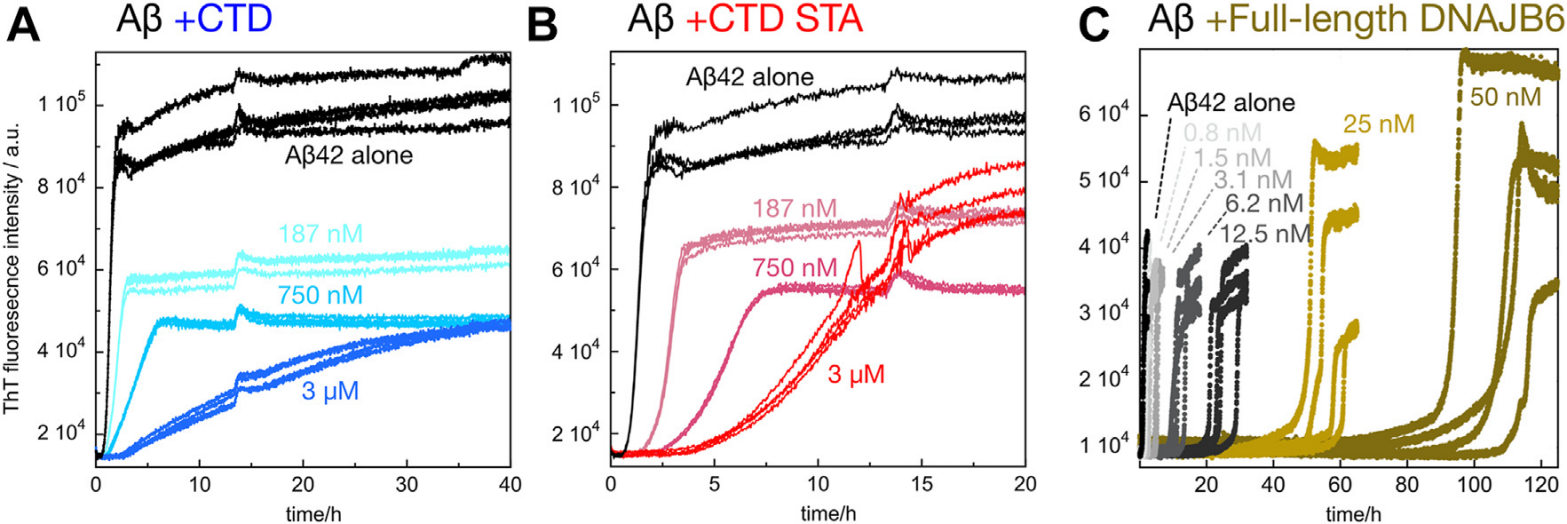
**Antiamyloid activity of C-terminal domain (CTD) constructs and DNAJB6.** Time-dependent measurements of thioflavin T (ThT) fluorescence to follow the aggregation of 3 μM Aβ42 alone (*black*) and in the presence of CTD (*blue shades*; (*A*), CTD STA (*red shades*; (*B*) or full-length DNAJB6 (*silver-gold*; (*C*) in 20 mM sodium phosphate, 0.2 mM EDTA, pH 8.0 with the color codes for the additive concentration given in each panel. The data in *C* are from Ref. (28). Aβ, amyloid-β.

The effect of CTD on Aβ42 amyloid formation was examined by studying the spontaneous time-dependent aggregation of highly pure recombinant Aβ42 *via* the fluorescence intensity of the amyloid-specific dye ThT. ThT increases its quantum yield upon binding to amyloid structures (30), which makes it suitable as a reporter probe for amyloid formation. A reaction half time (*t*_1/2_) of approximately 1.5 h was observed for a sample of 3 μM Aβ42, in agreement with previous data at similar conditions (19). The effect of CTD and CTD STA was evaluated by varying their concentration while keeping the Aβ42 concentration fixed at 3 μM. Addition of CTD or CTD STA at CTD:Aβ42 M ratios from 0.05:1 to 1:1 caused concentration-dependent retardation with the main effect being a change in slope of the transition (Fig. 3, *A* and *B*). CTD at a substochiometric 0.5:1 M ratio increased *t*_1/2_ by almost 10-fold compared with Aβ42 alone. Cryo-EM analyses of samples taken at the final plateau confirm the formation of fibrils in samples both without and with CTD (Fig. S6, *A* and *B*). The fibrils formed in the presence of CTD do, however, appear longer and thinner and with longer twist distance than those formed from Aβ42 alone. The effect of CTD STA is different from that of CTD with a combined effect on the length of the lag phase and the slope of the transition. Clearly, for both CTD and CTD STA, the effect on curve shape is very different, and the concentration ratio required for a significant effect is higher compared with full-length DNAJB6 (Fig. 3*C*). DNAJB6 causes a delay of the aggregation curve with retained steepness of the transition, and DNAJB6:Aβ42 M ratios of as little as 0.005 to 0.04:1 leads to 10- to 100-fold increases in the *t*_1/2_ at similar solution conditions as used here (5, 6, 31).

### Kinetic analysis

The ThT fluorescence intensity for all replicates at each concentration of each construct was normalized and included in the kinetic analysis using the integrated rate laws for fibril growth as described earlier (19, 29). Initial fitting to all data obtained in the presence of each construct was performed three times using as a single variable parameter either the rate constant for primary nucleation, *k*_n_, secondary nucleation, *k*_2_, or elongation, *k*_+_, based on Equation 1 with [M]_0_ = 0 (nonseeded experiments). In each of these three attempts, the remaining two parameters were fixed at the values previously obtained for Aβ42 alone (14). For CTD, we find that the analysis using *k*_2_ as a variable parameter fits the data significantly better than the other two options (*k*_n_ or *k*_+_ variable; Fig. S7). The improvement in the error square sum is a factor of 16 relative to *k*_n_ and a factor of 6 relative to *k*_+_. For CTD STA, the analysis using *k*_+_ as a variable parameter fits the data better than the other two options (*k*_n_ or *k*_2_ variable; Fig. S8). The improvement in the error square sum is a factor of 8 relative to *k*_n_ and a factor of 2 relative to *k*_2_. The kinetic analysis of data from nonseeded experiments is only reliant on the products *k*_+_ *k*_2_ and *k*_+_ *k*_n_ (Equations 2 and 3). Seeding experiments were therefore carried out in order to distinguish between effects on *k*_+_ and *k*_2_. Aggregation reactions starting from Aβ42 monomer plus preformed seeds at four defined seed concentrations (0.3, 1, 30, and 50% in monomer units) were used to probe the effect of each construct on mainly secondary nucleation (0.3 and 1% seed) or on mainly elongation (30 and 50% seed). The data obtained at low seed concentration show a clear retardation and change in slope of the transition, implying very clearly that the rate of secondary nucleation is reduced although the effect may be slightly lower for CTD STA compared with CTD (Fig. S9*A*). The data obtained at 50% seed show that there is an additional effect on elongation, most prominently not only for CTD STA but also for CTD (Fig. S9*B*). From the initial rate at 50% seed, we can estimate the effect on *k*_+_ for each construct as a function of its concentration.

**Figure 4:**
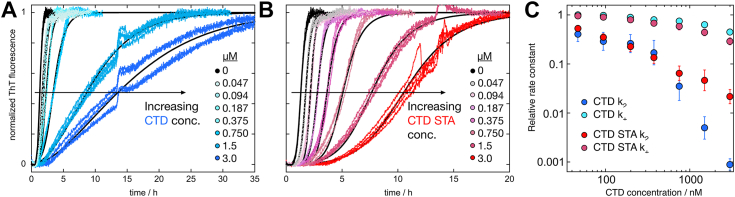
**Kinetic analyses of nonseeded data for Aβ42 in the presence of C-terminal domain (CTD).** Final analysis of data from nonseeded experiments for Aβ42 in the presence of CTD (*A* and *C*) or CTD STA (*B* and *C*), using the construct-concentration-dependent values of *k*_+_ from fits to data from 50%-seeded experiments and *k*_2_ as a fitted parameter. Fits to data are shown in (*A* and *B*), and the effect on *k*_+_ and *k*_2_ is shown in (*C*) as averages and standard deviation over 12 replicates at each inhibitor concentration. The data were obtained at 37 °C, whereas the results obtained at 15 °C are shown in Fig. S10. Aβ, amyloid-β.

A final kinetic analysis of data from nonseeded reactions was performed using the information from the analysis of the reactions with 50% seeds. In this analysis, we used the value of *k*_+_ at each construct concentration as a fixed curve-specific parameter, *k*_n_ as a fixed global parameter, and *k*_2_ as the curve-specific fitted parameter. This analysis provides an improved representation of the nonseeded data compared with variation of *k*_2_ alone and allows us to discern for each construct the relative effect on *k*_2_ and *k*_+_ as a function of construct concentration. This final analysis of the data from nonseeded experiments is shown in Figure 4 and is found to result in improved or much improved fits compared with the analysis with a single affected rate constant. There is no remaining discrepancy that would support an effect on *k*_n_ for any of the constructs.

For CTD, the fit with *k*_2_ as the single variable parameter is relatively good (Fig. S7), but the fit improves by using the variation in *k*_+_ as inferred from the 50% seed experiment (Fig. 4*A*). The effect on *k*_2_ clearly dominates over the effect on *k*_+_ (Fig. 4*C*); the effect on *k*_2_ is 100- to 1000-fold higher than the effect on *k*_+_ at the highest CTD concentrations tested (0.75–3 μM). The combined analysis with both nonseeded and seeded data thus confirms that for CTD the effect on secondary nucleation strongly dominates.

For CTD STA, the analysis with a single variable rate constant produces a slightly better fit to the data with variation of *k*_+_ compared with variation of *k*_2_ (Fig. S8); still none of these options are very good. The fit to the data improves significantly if we use the variation in *k*_+_ as inferred from the 50% seed experiment and then fit *k*_2_ to the data at each CTD STA concentration (Fig. 4*B*). The effects on *k*_+_ and *k*_2_ are compared in Figure 4*C*. The effect on *k*_2_ is ca. 10-fold higher than the effect on *k*_+_ at the three highest CTD STA concentrations tested (0.75–3 μM). The combined analysis thus implies that for CTD STA the effect on secondary nucleation dominates over the effect on elongation. Given that the effect of reduced *k*_2_ on the aggregation curve shape does not change very much beyond a 100-fold reduction and not at all after a 1000-fold reduction (32), the difference between CTD and CTD-STA at the highest three concentrations should be interpreted with caution.

The whole experiment was repeated three times at 37 °C, with four replicates of each inhibitor concentration in each repeat. The experiment was also repeated three times at 15 °C, with four replicates of each inhibitor concentration in each repeat, as both CTD and CTD STA are close to fully folded at this lower temperature (Fig. 2*C*). The data are shown in Fig. S10, *A* and *B* together with fits assuming selective reduction of secondary nucleation. These fits were motivated by the data under high seeding conditions (50% seed), which show no effect on the elongation rate of any of the two constructs at 15 °C (Fig. S10*C*). The effect of *k*_2_ is plotted *versus* inhibitor concentration in Fig. S10*D*.

### Interaction between CTD and Aβ42 aggregates

**Figure 5:**
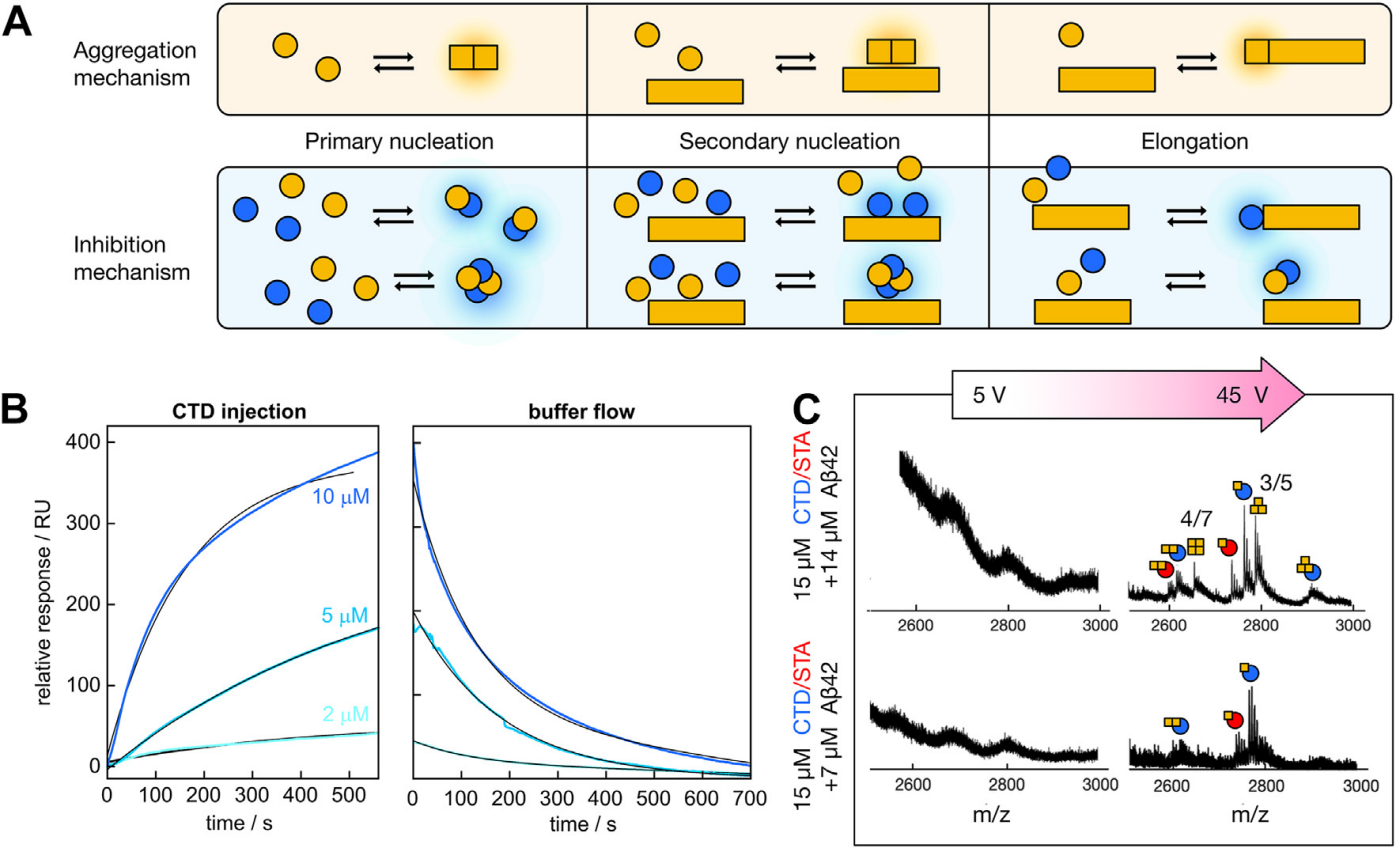
**Characterization of interaction between C-terminal domain (CTD) constructs and Aβ42.***A*, the microscopic steps in amyloid formation that are used to fit the aggregation data Aβ42 data (*top*). Primary nucleation produces nuclei through interactions between monomers only, secondary nucleation produces nuclei from monomers catalyzed by fibrils, and elongation involves the growth of existing fibrils by addition of monomers. Modulation of these steps can arise because of interactions with different species (*bottom*). The formation of co-oligomers could decrease the rate of not only primary nucleation of pure fibrils but also secondary nucleation. Blocking the fibril surface or ends could decrease secondary nucleation and elongation, respectively. *B*, surface plasmon resonance (SPR) data in 20 mM sodium phosphate, 0.2 mM EDTA, pH 8.0, 0.005% Tween-20, for the interaction of CTD with immobilized Aβ42 fibrils at 2 (*light blue*), 5 (*medium blue*), and 10 (*blue*) μM CTD (*left*) and the data obtained under buffer flow following the respective CTD injection (*right*). All data are shown with colors, and the fitted curves are shown in *black*. The corresponding SPR data for CTD STA are shown in Fig. S20. *C*, native MS data of Aβ42 that has been coincubated with CTD and CTD STA, measured at trap collision energies of 5 V (*left*) and 45 V (*right*). The homo Aβ42 oligomers are annotated by their oligomeric state/charge state (n/z) ratio. Heterooligomers are illustrated by symbols built up by Aβ42 (*yellow square*), CTD (*blue circle*), and CTD STA (*red circle*). Aβ, amyloid-β; MS, mass spectrometry.

Inhibition of secondary nucleation could be due to interactions with the amyloid fibril surface (Fig. 5*A*). The interaction between CTD and immobilized Aβ42 fibrils was studied using surface plasmon resonance (SPR) technology. Injection of CTD resulted in a change in refractive index close to the surface indicative of an interaction with the fibrils, and an association phase clearly dependent on the injected construct concentration was observed (Fig. 5*B*). The data are indicative of relatively fast exchange rates (see Table S1 for fitted parameters) and a moderate affinity with an equilibrium association constant *K* of around 10^5^ M^−1^. The data for CTD STA are highly similar to those for CTD, indicating that the ST/A substitutions do not affect the affinity for Aβ42 fibrils. No binding to a control surface without immobilized fibrils was observed. We also studied the possible complex formation between CTD and Aβ42 during an ongoing aggregation reaction using MS. Direct native MS analysis shows that coincubation of CTD with Aβ42 results in a noticeable decrease in the amount of observed CTD homodimers.

The occurrence of Aβ42–CTD complexes was also confirmed using native MS. A small fraction of Aβ42–CTD heterodimers could be detected. A relatively high collisional activation was, however, required to obtain sufficient signal intensity as well as to detect any larger Aβ42 oligomers or Aβ42–CTD coaggregates (Fig. 5*C*). By MS–MS, we did not detect any crosslinks between Aβ42 and CTD but between Aβ42 and the CTD within intact DNAJB6 (Fig. S11, *A*–*C* and Table S2). Both Aβ42–CTD and Aβ42–CTD STA heterooligomers were detected upon increased collisional activation, indicating that both variants may interact with Aβ42. The conditions are similar to those needed to detect Aβ42 oligomers or coaggregates upon coincubation with micelles (33, 34), indicating that declustering of larger coaggregates is taking place. This would point toward interactions between Aβ42 and CTD in higher order aggregation states instead of small heterooligomers.

### CTD segments grafted to S100G

**Figure 6:**
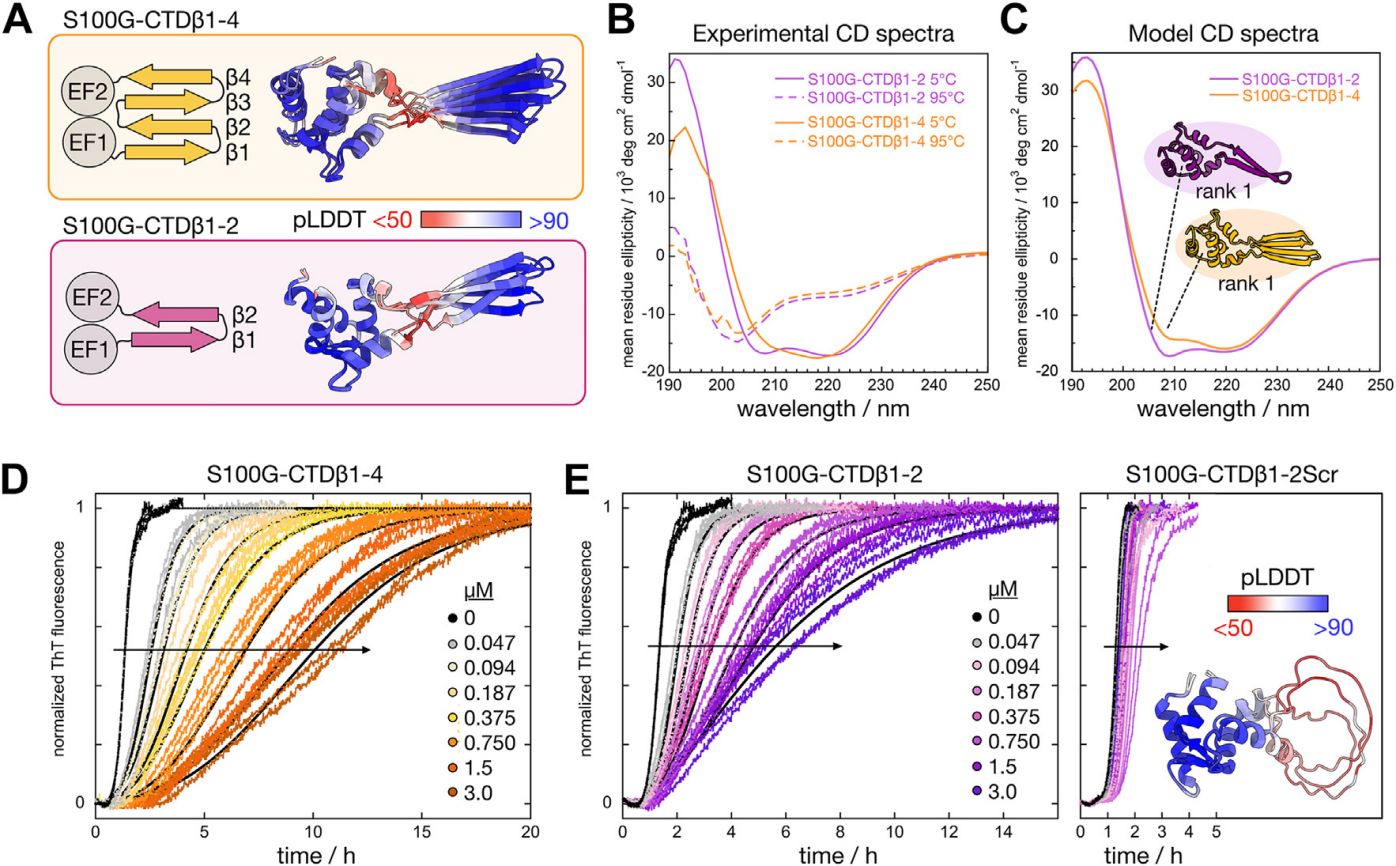
**Structure and antiamyloid activity of S100G-CTD constructs.***A*, architecture of S100G-CTDβ1–4 (*top*) and S100G-CTDβ1–2 (*bottom*) with the β-strands from CTD grafted between the two EF-hands of S100G. AlphaFold2 predictions (top three ranks) for the two constructs are shown colored according to their pLDDT scores (*red* to *blue*). *B*, experimental far-UV CD spectra of S100G-CTDβ1–2 (*purple*) and S100G-CTDβ1–4 (*orange*) at 5 °C (*solid line*) and at 95 °C (*dashed line*). *C*, predicted far-UV CD spectra for the top ranked AlphaFold2 models of S100G-CTDβ1–2 (*purple*) and S100G-CTDβ1–4 (*orange*), predicted using PDBMD2CD (https://pdbmd2cd.cryst.bbk.ac.uk). *D*, time-dependent ThT kinetics data for Aβ42 (3 μM) aggregation in the presence of increasing concentration of S100G-CTDβ1–4 in 20 mM sodium phosphate, 0.2 mM EDTA, pH 8.0. The fits are from the final analysis of data from nonseeded experiments using the construct-concentration-dependent values of *k*_+_ from fits to data from 50%-seeded experiments and *k*_2_ as a fitted parameter. *E*, time-dependent ThT kinetics data for Aβ42 (3 μM) aggregation in the presence of increasing concentration of S100G-CTDβ1–2 (*left*) or the S100G-CTDβ1–2Scr variant where the sequence of the grafted CTD segment in S100G-CTDβ1–2 has been scrambled (*right*) in 20 mM sodium phosphate, 0.2 mM EDTA, pH 8.0. The AlphaFold2 prediction (top three ranks) of the S100G-CTDβ1–2Scr construct is also shown colored according to their pLDDT scores (*red* to *blue*). For the data on S100G-CTDβ1–2, the fits are from the final analysis of data from nonseeded experiments using the construct-concentration-dependent values of *k*_+_ from fits to data from 50%-seeded experiments and *k*_2_ as a fitted parameter. CTD, C-terminal domain; ThT, thioflavin T.

In order to study the functional role of β-strand 1 in CTD, we produced constructs with smaller parts of the CTD. S100G-CTDβ1–2 contains β-strand 1 and 2 (residues 187–212) and S100G-CTDβ1–4 four β-strands (residues 187–231) grafted between the two EF-hands of human S100G (Fig. 6*A*). These constructs thus lack the very C-terminal end of the CTD and were produced as WT and the corresponding ST/A-substituted variants. AlphaFold2 predicts that the original β-sheet architecture may form between the two EF-hands (Fig. 6*A*), and the predicted structural elements are all in excellent agreement with the experimentally solved NMR structures for S100G (Fig. S1, *C* and *E*) and CTD (Fig. S1, *D* and *F*). Far-UV CD spectroscopy further confirms a folded and mostly helical structure, with more negative ellipticity at 220 nm compared with the parent protein S100G, which could be attributed to the grafted β-sheet segment from CTD (Fig. 6*B*). The experimental spectra are moreover in excellent agreement with theoretical CD spectra generated from the AlphaFold models (Fig. 6*C*), indicating that the inserted loops of the produced constructs have folded into their intended β-sheet structures. Refolding of S100G after temperature-induced unfolding is fully reversible, whereas the insertion of CTD segments makes refolding almost fully reversible for S100G-CTDβ1–2 and less reversible for S100G-CTDβ1–4 (Fig. S2). The data further imply that the insertions of the CTD β-sheets destabilize S100G. The S100G-grafted constructs are, however, more stable than CTD (Fig. S2). The dimerization of the S100G-grafted constructs was studied using chemical crosslinking and SDS-PAGE (Fig. S12, *A* and *B*). As aforementioned, an upper limit for the affinity can be estimated based on the integrated intensity of gel bands corresponding to monomers and dimers and the total concentration of each construct (10 μM). For S100G-CTDβ1–2, we estimate the dimer-to-monomer equilibrium dissociation constant (*K*_*D*_ = [monomer]^2^/[dimer]) to *K*_*D*_ ≥60 μM (=8^2^ μM/1). For S100G-CTDβ1–4, the dimer band is very weak, and we can only estimate an approximate *K*_*D*_ >500 μM (=9.6^2^ μM/0.2) meaning at least a factor of 8 lower dimerization affinity compared with S100G-CTDβ1–2. For the alanine-substituted grafts, the dimer band is extremely weak, thus *K*_*D*_ appears to be at least 1 mM. MS–MS analysis of S100G-CTDβ1–2 identified the K189–K189 crosslink in the formed dimers (Fig. S12*C*).

S100G is a Ca^2+^-binding protein, also known as calbindin D_9k_, with one Ca^2+^-binding site in each EF-hand, which are energetically coupled, resulting in a positive cooperativity of Ca^2+^ binding. The destabilizing effect of grafting the CTD β-strands could thus also be studied by measuring the Ca^2+^-binding affinity and cooperativity of the constructs (Fig. S13). The larger graft in S100G-CTDβ1–4 caused a minor perturbation of the Ca^2+^-binding function. The data follow a sigmoidal trend of the same sign as the parent protein but more shallow. Fitting Supplementary Equations 8 and 9 to the data reveals that the average Ca^2+^ affinity (average lgK_av_ = 8.0) is within a factor of 2 from S100G (average lgK_av_ = 8.3), but the level of positive cooperativity seems to be reduced (−ΔΔG_η=1_ = 1.4 kJ/mol) compared with S100G (−ΔΔG_η=1_ = 9 kJ/mol). The smaller graft in S100G-CTDβ1–2 l caused a major perturbation of Ca^2+^ binding. While the affinity (average lgK_av_ = 7.8) is only reduced by a factor of 3, the affinity for the first-bound Ca^2+^ ion (lgK_1_ = 8.5) is significantly higher than for the second-bound Ca^2+^ ion (lgK_2_ = 7.0) implying that the positive cooperativity is lost. Thus, for both grafts, the effect on the average Ca^2+^ affinity is small, but the positive cooperativity of binding is reduced, implying that the communication between the two EF-hands is affected by insertion of the β-strands.

### Antiamyloid effect of the β-sheet grafts

Both S100G-CTDβ1–2 and S100G-CTDβ1–4 as well as their corresponding ST/A-substituted variants display antiamyloid activity observed as a prolonged Aβ42 aggregation time in ThT kinetics experiments. Especially, the slope of the transition is reduced, and the data are best fitted assuming a reduction of the rate constant for secondary nucleation (Figs. 6, *D* and *E* and S14–S17). Cryo-EM analyses of samples taken at the final plateau confirm the formation of fibrils in samples with S100G-CTDβ1–2 or S100G-CTDβ1–4 (Fig. S6, *C* and *D*). The Aβ42 aggregation kinetics in the presence of an S100G control protein was also measured in order to test if this antiamyloid effect is due to the grafted β-strands from DNAJB6 or simply an effect of the S100G scaffold. It is known that globular proteins can delay amyloid aggregation by nonspecific interactions (35) and that these effects have a strong charge dependence (36). Therefore, an S100G variant with a net charge of −4 at pH 8, because of the mutations E17Q + D19N + E26Q, was used. This can be compared with the net charge of −2 for S100G-CTDβ1–2 and −5 for S100G-CTDβ1–4 at pH 8. The effect of two positively charged proteins scMn+8 (net charge +8) (37) and chicken lysozyme (net charge +9) were also studied. The uneven charge distribution in S100G-CTDβ1–2 prompted the investigation of a second control protein, S100G-CTDβ1–2Scr with the same amino acid residue composition as S100G-CTDβ1–2 but with the sequence of the inserted β1–2 loop scrambled (Fig. S1). AlphaFold prediction yielded a structure model of S100G-CTDβ1–2Scr where the inserted scrambled loop has the lowest possible prediction score (<50) and is modeled as an unstructured segment (Fig. 6*E*). Low prediction scores in AlphaFold have been shown to be an indicator of disorder (38), and the prediction of an unfolded loop was confirmed using CD spectroscopy where the experimental spectrum agreed nicely with the predicted spectrum for the AlphaFold model (Fig. S2*F*). The S100G-CTDβ1–2Scr displayed only a minor antiamyloid activity (Fig. 6*F*), whereas the positively charged control proteins displayed a strong retardation and the S100G -4 variant displayed no activity (Fig. S18). This indicates that the antiamyloid activity of the two S100G-CTD constructs is likely because of the folded β-strands from DNAJB6, rather than because of S100G or nonspecific protein charge effects. The similar effects of the two S100G-CTD constructs indicate further that the first β-hairpin (β1 and β2) is sufficient for the antiamyloid activity observed for the CTD.

The effects on the ThT kinetics by the S100G-grafted constructs are qualitatively similar to the effects observed for CTD in that the main effect is a reduced slope of the transition. The concentration-dependent reduction of the slope, and the resulting increase in *t*_1/2_ is, however, not as pronounced as for CTD. Fitting to the data, in the same way as described for aforementioned CTD, reveals that the antiamyloid activity is mostly because of inhibition of secondary nucleation, with an additional small effect on the elongation rate in the case of S100G-CTDβ1–4 (Figs. S14–S17). The data together with the final fit to data from nonseeded reactions using information from seeded ones are shown in Figure 6*D* for S100G-CTDβ1–4 and in Figure 6*E* for S100G-CTDβ1–2. Binding of the S100G-grafted constructs to Aβ42 fibrils was observed using SPR (Fig. S19). The affinities of S100G-CTDβ1–2 and S100G-CTDβ1–4 for the fibrils were 3 to 100 times higher than for CTD, with equilibrium constant, *K*, of 3 × 10^5^ M^−1^ and 1 × 10^7^ M^−1^, respectively. The data for S100G-CTDβ1–4STA are highly similar to those for S100G-CTDβ1–4, and the data for S100G-CTDβ1–2STA are highly similar to those for S100G-CTDβ1–4. No binding to control surfaces with immobilized monomers, or no immobilized protein, was observed. The fitted parameters are found in Table S1.

## Discussion

Protein aggregation and amyloid formation are occurring continuously in living systems. The maintenance of proteostasis (protein homeostasis) requires many chaperones and protein quality control systems that uphold the delicate balance between protein synthesis, folding, and degradation (39, 40). When proteostasis is declining in performance, synthesized proteins may not fold efficiently, metastable proteins lose their functionally active conformations, and cytotoxic protein aggregates accumulate, which is associated with many age-dependent neurodegenerative diseases. In case of Alzheimer's disease, associated with the amyloid formation of Aβ peptide herein investigated, recent data from complex *in vivo* studies in new neuronal model systems suggest lysosomal quality control deficits in diseased neurons (41, 42). A number of different chaperones may inhibit amyloid formation (1, 2, 3, 4, 5, 6, 7, 8, 9, 10, 11, 43, 44, 45, 46, 47). The mechanism of amyloid formation has been extensively studied *in vitro* for several proteins, revealing a conformity in terms of underlying steps; in principle, for all systems studied, there appears to be primary and secondary nucleation, elongation, and in some cases, also fragmentation. The relative rate constants of these steps and thereby step dominance varies between systems, yet the set of steps is retained, for example, in the case of Aβ42 in buffer (14) and in CSF (26). While studies of amyloid mechanisms *in vivo* are emerging (48, 49, 50), a detailed investigation *in vitro* can provide information of which steps are affected by an inhibitor. Parallel studies of a set of antibodies revealed for each antibody that the same steps are affected in buffer and CSF (27).

### Shift of inhibitory mechanism

The results of the current study imply that the isolated CTD of the DNAJB6 chaperone, or constructs containing β-strands 1 and 2 from this domain, display antiamyloid activity toward Aβ42 aggregation *in vitro*. This activity is, however, different from that of full-length DNAJB6, with a distinct shift of inhibition mechanism. DNAJB6 strongly reduces the rate of primary nucleation and has an effect also on the rate of secondary nucleation (5), leading to a very large extension of the lag phase. In contrast, all three constructs studied here—and their respective STA controls—seem to mainly decrease the rate of secondary nucleation, leading to a reduced slope of the transition. The inhibition of secondary nucleation by the CTD is approximately as efficient as previously observed with the Brichos domain (47) and with fibril-binding proteins derived by phage display (32). Some of the constructs also show an additional small effect on elongation seen as a minor extension of the lag phase and a change in curve shape.

It should be noted that full-length DNAJB6 decreases the rate of secondary nucleation to approximately the same extent as the CTD at low client:chaperone ratios (5), indicating that the CTD has lost the ability of the full-length proteins to inhibit primary nucleation but retained its ability to inhibit secondary nucleation. This suggests that another part of DNAJB6 is responsible for primary nucleation inhibition, or that synergistic effects between CTD and other parts of DNAJB6 are required for such activity.

### Altered mode of interaction

Potent inhibition of Aβ42 aggregation is a feature observed for many chaperones (5, 6, 44, 47, 51). A shift in mechanism, in terms of which microscopic step(s) are modulated by an inhibitor, may be related to a shift in the mode of interaction, in terms of which species the chaperon or domain interact with (Fig. 5*A*; (52, 53)). Interactions with monomers may lead to inhibition of not only primary nucleation but also secondary nucleation and elongation because the monomer is a reactant in these steps as well. Interactions with oligomers, or the formation of co-oligomers at the expense of pure amyloid peptide oligomers, may lead to inhibition of not only primary nucleation but also secondary nucleation. Interactions with fibril surfaces may block the catalytic sites for secondary nucleation, and interactions with fibril ends may interfere with elongation. For DNAJB6, an interaction with Aβ42 oligomers or the formation of co-oligomers at the expense of pure Aβ42 oligomers may explain its potency to inhibit primary nucleation of Aβ42 fibrils (5, 6). The current SPR and native MS results suggest that CTD, as well as the S100G with grafted segments from CTD, interact with fibrils but not with small oligomers of Aβ42. The observed inhibition of Aβ42 secondary nucleation by CTD and the grafts is therefore most likely because of an interference with the catalysis at the fibril surface. The current data thus suggest that the inhibition of secondary nucleation by DNAJB6 can be ascribed to its CTD, whereas the inhibition of primary nucleation is a feature of the rest or the whole protein.

### The role of the Ser/Thr to Ala substitutions, CTD stability, and dimerization

The five STA mutations were incorporated in CTD as well as in the grafts S100G-CTDβ1–2 and S100G-CTDβ1–4 because these five substitutions have been found to reduce the potency of DNAJB6 in the inhibition of primary nucleation (5, 6, 31). However, these substitutions have less effect on the potency of CTD to inhibit secondary nucleation, suggesting that the role of Ser190, 192, 194 and Thr 193, 195 in the first β-strand of CTD is not the same in the inhibition of primary and secondary pathways.

The thermal denaturation midpoint, *T*_m,_ is reduced by 19 °C, from 58 °C for CTD to 39 °C for the STA variant, and the CD spectra indicate that CTD STA on average contains less β-sheet than CTD, reflecting a larger population of unfolded protein (Fig. 2, *B* and *C*). This is corroborated by the native mass spectra, which show an increased population of highly charged states for CTD STA compared with CTD. The replacement of five hydrophilic side chains with the hydrophobic alanine thus favors the unfolded over the folded form, meaning alanine either destabilizes the folded form or stabilizes the unfolded form of CTD. The shift in stability may reflect a reduced equilibrium constant for dimerization, which is energetically coupled to the folding–unfolding equilibrium. The crosslinking experiments indeed reveal a reduced population of dimers for CTD STA compared with CTD, implying a reduced monomer–dimer equilibrium constant for the mutant. From the fitted denaturation parameters (Fig. 2, *B* and *C*), we can estimate that the fraction of unfolded protein is ca. 5% for CTD and 45% for CTD STA at the temperature of the aggregation experiments (37 °C). The kinetic analysis suggests that CTD has a small effect also on elongation, and this effect is more prominent for CTD STA, although for both variants, the effect on secondary nucleation dominates (Fig. 4). It is thus possible that the folded CTD interacts with fibril surfaces blocking secondary nucleation and the unfolded monomers interact with fibril ends reducing the rate of elongation. The fibril end exposes the hydrophobic core of the terminal monomer plane, which may explain the increased effect on elongation of the more hydrophobic CTD STA compared with CTD. This interpretation is strengthened by the data at 15 °C, under which condition, both variants are close to fully folded, no effect on elongation is found, and both variants exclusively inhibit secondary nucleation (Fig. S10). At 15 °C, the aggregation of Aβ42 is even more dominated by secondary nucleation compared with 37 °C (15); it thus emerges that CTD STA may be a more effective inhibitor of secondary nucleation than CTD, in clear contrast to WT DNAJB6 being a more effective inhibitor of primary nucleation than DNAJB6 with the same STA substitutions (6).

### The role of CTD net charge

Many proteins have been found to delay amyloid aggregation (35), and these effects have a strong charge dependence with stronger inhibition the higher the net charge, that is, more positive for positively charged proteins and less negative for negatively charged proteins (36). The CTD (and CTD STA) monomer has a net charge of +3 over 56 residues at pH 8.0 (assuming charged ends), that is, +0.5 per kDa. It is therefore relevant to ask whether fibril binding and the effect on secondary nucleation is a specific feature of CTD, or whether this activity is a consequence of its positive net charge. The single-chain variant of the plant protein monellin with net charge +8 (scMN+8; +0.7 per kDa) (37) and chicken lysozyme with net charge +0.5 per kDa are both very potent inhibitors of Aβ42 aggregation at the same series of concentrations as used for CTD and CTD STA (Fig. S18). Indeed, the effect of lysozyme, with the same charge density, is indistinguishable from that of CTD, whereas the more highly charged scMn+8 is even more retarding. The large antiamyloid effect of these control proteins raises intriguing questions regarding the uneven charge distribution in DNAJB6. Since the net charge of DNAJB6 is close to zero around pH 8, its charge distribution around this pH can be estimated using a titration profile based on model p*K*_a_ values and ideal titration curves (54) (Fig. S21). While the total number of charged residues is high in both globular domains, they are predicted to carry a few units of net positive charge at pH 8 and connected by a negatively charged linker leading to an overall charge close to zero.

The S100G variant with net charge −4 displays no antiamyloid activity (Fig. S18), indicating that the effects of S100G-CTDβ1–4 (net charge −5) and S100G-CTDβ1–2 (net charge −2) are due to the β-strands of CTD rather than nonspecific protein charge effects. This −4 variant has a relatively uniform charge distribution over one domain, whereas each of S100G-CTDβ1–4 and S100G-CTDβ1–2 has one negative (−7) and one positive (+2 or +5) domain. Polarized charge interactions could therefore be possible in these cases. However, the S100G-CTDβ1–2Scr variant displayed very low antiamyloid activity (Fig. 6*E*). This variant has the same charge polarization as S100G-CTDβ1–2 but does not form the folded DNAJB6 β-hairpin structure as the sequence is scrambled. This indicates that the observed effects on amyloid aggregation by the CTD constructs are due to specific interactions involving the CTD β-sheet, rather than nonspecific charge effects.

### The role of β-strand 1 in CTD

AlphaFold predicts that the interaction between two CTD monomers in the homodimer involves the two copies of β-strand 1 from the two monomers in an antiparallel arrangement leading to a 10-stranded β-sheet involving both monomers (Fig. S4). While the large distance allowance (30 Å) of the crosslinker used could in principle link any pair of lysines in a dimer, the K189–K189 crosslink clearly dominates (Fig. 2*D* and Table 1), implying that this pair of lysine side chains reacts more easily than other pairs in the formed dimer. This is corroborated by the AlphaFold prediction, in which the two K189 side chains are indeed on the same face of the 10-stranded β-sheet, whereas K189 in one monomer and K196 in the other monomer, although as close in space, are placed on opposite faces of the sheet (Fig. S4). A corresponding crosslink analysis of S100G-CTDβ1–2 again identified the K189–K189 crosslink in the formed dimers (Fig. S12*C*). The apparently lower dimer affinity for S100G-CTDβ1–4 compared with S100G-CTDβ1–2 could have many molecular origins, and the small free energy difference (ΔΔG ≥6 kJ/mol) may indeed not be larger than what is seen upon removal of a single methylene group at a hydrophobic interface (55).

β-strand 1 in CTD has been hypothesized to interact with Aβ based on reduced inhibitory potency of full-length DNAJ6 with substitutions in this strand (6). Contacts between β-strand 1 in CTD and Aβ42 are predicted by an AlphaFold model of a CTD-Aβ42 heterodimer (Fig. 5*D*) and is detected in crosslinking experiments with full-length DNAJB6 but not with the CTD construct (Table S2). The similar effects on secondary nucleation by S100G-CTDβ1–2 and S100G-CTDβ1–4, and the lack of effect by S100G-CTDβ1–2Scr, imply that β-strands 1 and 2 are sufficient for the antiamyloid effect in the grafts.

### The role of chaperone chemical potential

The discrepancy between the large effect of the STA substitutions in β-stand 1 of DNAJB6 on the one hand (5, 31) and the lack of effect of the same substitutions in CTD or the grafts on the other hand raises the question of whether the five Ser and Thr residues play additional roles than in direct interaction with client peptides. An alternative mechanism behind the antiamyloid activity and suppression of primary nucleation of Aβ42 fibril formation by DNAJB6 may be an unusually high chemical potential of the chaperone (56). In such mechanism, the chemical potential of the chaperone would be significantly reduced upon formation of coaggregates with Aβ42, in which the amyloid peptide may gain a higher chemical potential compared with pure amyloid fibrils. This would cause a shift in the amyloid formation equilibrium toward a higher solubility of Aβ42 (56), as is indeed observed (6). The fact that the five S/T substitutions make DNAJB6 a less potent inhibitor of primary nucleation and a less efficient enhancer of Aβ42 solubility suggests that these substitutions may potentially affect the antiamyloid function of DNAJB6 by lowering its chemical potential, thus making it less prone to form coaggregates with client peptides. In contrast, CTD and the two grafted constructs seem to behave more like regular folded proteins, implying that the high chemical potential of DNAJB6 is either a feature of the entire protein or of the missing unstructured linker and/or JD. This is supported by the observation that the nonoligomeric DNAJB6 ΔS/T variant affects Aβ42 aggregation in qualitatively similar ways as CTD, with a decrease in the rate constant for secondary nucleation without a significant change in primary nucleation (6).

### The role of chaperone oligomers

Like amyloid proteins (57), many chaperones are prone to self-assembly. There is an intriguing correlation between oligomerization of DNAJB6 and its potency as an antiamyloid chaperone. For example, substitutions that render the chaperone less oligomeric also interfere with its effect on aggregation kinetics and amyloid peptide solubility (6, 22). However, DNAJB6 is active as a chaperone at concentrations well below its critical aggregation concentration. Ultrapure DNAJB6 preparations may for example significantly inhibit the aggregation of 3 μM Aβ42 at 15 nM DNAJB6 (10-fold increase in *t*_1/2_) (31), whereas the critical aggregation concentration was recently found to be around 100 nM (58). Oligomerization and amyloid inhibition may thus be two consequences of the same chaperone property. This property may be a high chemical potential of DNAJB6, which it can lower through coassembly with client peptides or through self-assembly with other copies of itself. The extreme polydispersity suggests that the chemical potential is high in oligomers of all aggregation numbers, that is, no self-assembly arrangement is significantly more stable than another one (56). The five S/T substitutions may potentially lower the chemical potential of DNAJB6, thus making it less prone both to coassemble with other proteins and to self-assemble. No large oligomers are observed for CTD, which in addition to monomers, is observed as a low population of dimers and tetramers reliant on the S/T residues in β-strand 1 (Fig. 2, *E* and *F*). This implies that the formation of large oligomers is reliant on other parts of DNAJB6 than CTD alone, as was aforementioned also concluded for its capacity to inhibit primary nucleation. The high chemical potential of DNAJB6 thus seems to be a feature governed by other parts of the full-length protein or a synergistic effect of both folded domains and the linkers.

## Conclusions

The strong potency of DNAJB6 to inhibit primary nucleation at substoichiometric molar ratio is not seen with CTD or any of the S100G-CTD constructs, which, however, reduce the rate of secondary nucleation to a similar extent as DNAJB6. The formation of transient co-oligomers as between Aβ42 and DNAJB6 is not observed between Aβ42 and CTD or the S100G-CTD constructs. A third aspect of DNAJB6 not replicated by CTD or the S100G-CTD constructs is the formation of large and polydisperse oligomers. CTD forms dimers only weakly (*K* = 3 × 10^4^ M^−1^) and even weaker if five S/T residues in β-strand 1 are mutated to A. The shift in inhibitory mechanism seems to be due to binding of CTD to Aβ fibrils rather than oligomers, and fibril binding is apparently not reliant on these S/T residues. The inhibition of primary nucleation and the formation of large oligomers are thus two properties of DNAJB6, which are governed by other parts than CTD, or a synergistic effect of the entire DNAJB6 with its two folded domains and long unstructured linkers. Binding to Aβ42 fibrils and inhibition of secondary nucleation can, however, be mimicked by the isolated CTD or even a construct that only contains the first two β-strands of the CTD, which affects the nucleation events at the fibril surface. The results of the current study thus show that the retardation of secondary nucleation by DNAJB6 can be ascribed to its CTD, whereas the inhibition of primary nucleation is dependent on the rest or all the protein.

## Experimental procedures

All methods are described in detail in the Supporting information section.

### Prediction of structures using AlphaFold

All predictions were generated using the online version AlphaFold2 with MMseqs2, no templates, within ColabFold (59). A pLDDT value <50 indicates poor prediction, and pLDDT >90 corresponds to high confidence (59, 60).

### Expression and purification of DNAJB6 constructs

The sequences of the DNAJB6-derived proteins are found in Fig. S1. All proteins were purified using sonication, boiling, ion exchange, and SEC, as further described in the Supporting information section. Chromatograms and SDS-PAGE gels from the purification procedures are found in Fig. S22 (CTD), Fig. S23 (S100G-CTDβ1–2), and Fig. S24 (S100G-CTDβ1–4). The purity of all constructs was >99% based on the absence of other bands in overloaded Coomassie-stained SDS-PAGE gels; no other peaks were observed in SEC chromatogram and MALDI mass spectra.

### Expression and purification of Aβ42

Aβ(M1-42), here called Aβ42, was expressed and purified as described (14, 61) and stored as lyophilized aliquots after monomer isolation. The purity of Aβ42 was >99.5% based on the absence of other bands seen on silver-stained SDS-PAGE gel, the absence of signals from molecules in an ^1^H NMR spectrum; no other peaks were seen by SEC or in the MALDI mass spectrum. Monomers for the kinetics experiments were isolated from such aliquots using SEC 20 mM sodium phosphate, 0.2 mM EDTA, pH 8.0.

### Expression and purification of S100G (E17Q + D19N + E26Q), scMn+8, and lysozyme

The S100G mutant E17Q + D19N + E26Q and scMn+8 were expressed and purified as described (62, 63). The purity was >99.5% based on the absence of other bands seen on silver-stained SDS-PAGE gels, the absence of signals from molecules in an ^1^H NMR spectra; no other peaks were seen by SEC or in MALDI mass spectra. Chicken egg lysozyme was purchased from Sigma–Aldrich (NR) and purified by passing through an anion exchange resin to remove anionic contaminants. The purity was >99% based on the absence of other bands in overloaded Coomassie-stained SDS-PAGE gels, and no other peaks were observed by SEC. Each protein was subjected to SEC on Superdex 75 column to isolate pure monomer in 20 mM sodium phosphate, 0.2 mM EDTA, pH 8.0 prior to use in the kinetic assays.

### Native MS

Samples for native MS were buffer exchanged into 200 mM ammonium acetate (pH 6.8) to final protein concentrations of 10 to 25 μM. Aβ42 and CTD constructs were coincubated for 10 min at 37 °C prior to native MS analysis. Cross-section measurements were obtained from calibration, with reference cross-section values obtained from the literature (64).

### Chemical crosslinking MS

Samples were crosslinked and prepared for mass spectrometric analysis as described (18). Data acquisition and analysis was done as described (18), with details in the Supporting information section.

### CD spectroscopy and thermal denaturation

CD spectra and thermal scans were recorded using a Jasco J-815 spectropolarimeter as described in the Supporting information section.

### Prediction of CD spectra

Theoretical CD spectra were generated of the S100G-CTD constructs using the PDBMD2CD online software (65), as described in the Supporting information section.

### Nonseeded and seeded aggregation kinetics

Aggregation kinetics experiments were set up in 96-well PEG-ylated polystyrene plates, black with transparent bottom (Corning; catalog no.: 3881) in 20 mM sodium phosphate, 0.2 mM EDTA, pH 8.0.

### Kinetic analyses

Kinetic analyses of data were performed using the AmyloFit (amylofit.com) online software (66) and the following master equation that describes the time evolution of the fibril mass concentration, M.

(1) $$\frac{\left[M\right]}{{\left[M\right]}_{\infty }}=1-\left(1-\frac{{\left[M\right]}_{0}}{{\left[M\right]}_{\infty }}\right)*{\left(\frac{B_{+}+C_{+}}{B_{-}+C_{+}}*\frac{B_{-}+C_{+}e^{\kappa t}}{B_{+}+C_{+}e^{\kappa t}}\right)}^{\frac{k_{\infty }^{2}}{{\bar{k}}_{\infty }\kappa }}e^{-k_{\infty }t}$$

where the parameters are defined as follows:

(2) $$\kappa =\sqrt{2k_{+}k_{2}{\left[m\right]}_{0}^{n_{2}+1}}$$

(3) $$\lambda =\sqrt{2k_{+}k_{n}{\left[m\right]}_{0}^{n_{c}}}$$

(4) $$C_{\pm }=\frac{k_{+}{\left[P\right]}_{0}}{\kappa }\pm \frac{k_{+}{\left[M\right]}_{0}}{2{\left[m\right]}_{0}k_{+}}\pm \frac{\lambda^{2}}{\left({2\kappa }^{2}\right)}$$

(5) $$k_{\infty }=\sqrt{\frac{2\kappa^{2}}{[n_{2}\left(n_{2}+1\right)}+\frac{{2\lambda }^{2}}{n_{C}}}$$

(6) $${\bar{k}}_{\infty }=\sqrt{k_{\infty }^{2}-4C_{+}C_{-}\kappa^{2}}$$

(7) $$B_{\pm }=\frac{k_{\infty }\pm {\bar{k}}_{\infty }}{2\kappa }$$

In these relations, [m]_0_ is the initial monomer concentration, [P]_0_ and [P]_∞_ are aggregate number concentrations at the start of the reaction and after reaction completion, respectively. [M]_0_ and [M]_∞_ are the mass concentrations of fibrils at the start and end of the reaction, respectively. *k*_n_, *k*_2_, and *k*_+_ are the rate constants for primary nucleation, secondary nucleation, and elongation, respectively. n_c_ and n_2_ are the monomer reaction orders of primary and secondary nucleation, respectively. The values n_c_ = 2 and n_2_ = 2 were fixed based on previous results for Aβ42 (14).

### Cryo-transmission electron microscopy

Cryo-transmission electron microscopy images were acquired using a JEM 2200FS electron microscope.

### Ca^2+^ binding

The macroscopic Ca^2+^-binding constants, *K*_1_ and *K*_2_, for S100G-CTDβ1–4 and S100G-CTDβ1–2 were determined from competitive Ca^2+^ titrations *versus* the chromophoric Ca^2+^ chelator Quin2 in 2 mM Tris–HCl, pH 7.5. The data were fitted as described in the Supporting information section.

## Data availability

All data are contained within the article.

## Supporting information

This article contains supporting information (59).

## Conflict of interest

S. L. is a founder and employee of Wren Therapeutics Ltd. The authors declare that they have no conflicts of interest with the contents of this article.
