# Supplementary material for: The C-terminal domain of the antiamyloid chaperone DNAJB6 binds to amyloid-β peptide fibrils and inhibits secondary nucleation
Source: J Biol Chem. 2023 Oct 4;299(11):105317. doi: 10.1016/j.jbc.2023.105317 (PMC10641233; doi:10.1016/j.jbc.2023.105317)
Supplement: Supporting information [file mmc1.pdf]

Supporting information for

The C-terminal domain of the anti-amyloid chaperone DNAJB6 binds to Amyloid-β peptide fibrils and inhibits secondary nucleation

Nicklas Österlund[a*], Rebecca Frankel[b], Andreas Carlsson[b], Dev Thacker[b], Maja Karlsson[b], Vanessa Matus[b], Astrid Gräslund[a], Cecilia Emanuelsson[b], Sara Linse[b*].

[a]Department of Biochemistry and Biophysics, Stockholm University, 106 91 Stockholm, Sweden; [b]Biochemistry and Structural Biology, Department of Chemistry, Lund University, 221 00 Lund, Sweden.

*To whom correspondence may be addressed. Email: nicklas.osterlund@dbb.su.se or sara.linse@biochemistry.lu.se.

Supporting Information Text

Extended Materials and Methods

Prediction of structures using AlphaFold. All predictions in AlphaFold were generated using the online version of AlphaFold within ColabFold: AlphaFold2 w/ MMseqs2, and no templates were used [1]. AlphaFold2 uses a scoring called Predicted Local Distance Difference Test, pLDDT, to report the confidence of the predictions over the structure. A pLDDT <50 indicates that this position in the structure is not well predicted, thus it may be a flexible/unstructured part or for other reasons difficult to predict. pLDDT values > 90 correspond to high confidence [1,2]. CTD and CTD-STA were modelled as monomers and dimers, while S100G-CTDβ1-2, S100G-CTDβ1-4, and S100G-CTDβ1-2Scr were modelled as monomers. A complex between CTD and Aβ42 was predicted as a heterodimer. We also attempted to predict a structure for a heterodimer of Aβ42 and DNAJB6, but the lowest possible prediction score (<50) was obtained for the entire Aβ42 peptide, thus no results will be reported in this case.

Expression of DNAJB6 constructs. The sequences of the studied DNAJB6-derived proteins are found in Supporting Figure S1. All constructs were expressed from synthetic genes with E. coli-preferred codons cloned into the Pet3a vector (purchased from Genscript, Piscataway, New Jersey). Single colonies were used to inoculate 50 mL day cultures in LB medium with 50 mg/L ampicillin, and 30 mg/L chloramphenicol, grown in 250 mL baffled flasks at 37°C with continuous 125 rpm shaking. The equivalent of 0.5 mL at OD600 = 0.8 was transferred to 500 mL overnight auto-induction medium (2.5 mM Na_2HPO_4, 2.5 mM KH_2PO_4, 12 mM $(NH_4)_2SO_4$, 1 mM $MgSO_4$, 0.1 g/L glucose, 0.4 g/L lactose, 1 g/L glycerol, 10 g/L NaCl, 10 g/L tryptone, 5 g/L Bacto yeast extract, 50 mg/L ampicillin, 30 mg/L chloramphenicol) and cultures were grown in 2500 mL baffled flasks at 37°C with continuous 125 rpm shaking for 15 h. Cells were harvested by centrifugation at 6000 rpm in a JLA8.1000 rotor for 10 minutes. Cells were stored frozen until purification.

Purification of DNAJB6 C-terminal domains CTD and CTD STA. Cell pellet from 0.5 L was sonicated (2-4 min, 50 % duty cycle) in 60 mL of 15 mM Tris/HCl, 3 mM EDTA, pH 8.0 with a trace of DNAse. The sonicate was poured into 120 mL boiling water in an Erlenmeyer flask immersed in a boiling water bath, to immediately reach 70°C, and then heated up to 100 ºC under stirring. Precipitation of E. coli proteins was clearly visible. The flask was moved to an ice-water slurry and cooled under stirring until the temperature reached 15 ºC. A trace of DNAse was added and the solution was centrifuged for 10 min at 18000 rpm in a 25.50 rotor at 4ºC. The supernatant was collected and again centrifuged for 10 min at 18000 rpm in a 25.50 rotor at 4ºC. The clarified supernatant was passed through 20 mL anion exchanger in the form of Q Sepharose big beads in batch mode equilibrated in 5 mM Tris/HCl, 1 mM EDTA, pH 8.0 (buffer A). The flowthrough was loaded on a cation exchange column (20 mL SPHP, equilibrated in buffer A). The column washed

with 100 mL buffer A. CTD and CTD STA were eluted with a linear salt gradient from 0-200 mM NaCl in buffer A and fractions of 7 mL collected, total gradient volume 500 mL. The absorbance at 214 and 256 nm was recorded during elution and fractions were examined by SDS PAGE (10-20% Tris-tricine gels stained with Coomassie). Fractions 21-37 (Supporting Figure S20) were frozen (in 5 mM Tris/HCl, 1 mM EDTA, and ca. 70 mM NaCl). Peak fractions were purified by SEC on Superdex 75 (26/600 mm) in 20 mM sodium phosphate, 0.2 mM EDTA, pH 8.0, with the absorbance at 214 and 256 nm monitored (Supporting Figure S20). The concentration was estimated from the absorbance at 214 and 256 nm of the collected fractions, which were aliquoted and frozen. Samples for CD spectroscopy were prepared by SEC in 20 mM sodium phosphate, pH 8.0.

Purification of S100G-grafted DNAJB6 fragments. Cell pellet from 0.5 L was sonicated (2-4 min, 50 % duty cycle) in 60 mL of 10 mM Tris/HCl, pH 8.0 with a trace of DNAse. The sonicate was poured into 120 mL boiling buffer (60 mL of 10 mM Tris/HCl, pH 8.0) in an Erlenmeyer flask immersed in a boiling water bath, to immediately reach 70°C. The solution was heated up to 100 ºC under stirring and the precipitation of E. coli proteins was clearly visible. Then flask was moved to an ice-water slurry, 3 mM EDTA added from a concentrated stock (100 mM EDTA, pH 8.0) and the solution cooled under continuous stirring until the temperature was below 15 ºC. A trace of DNAse was added and the sample centrifuged for 10 min at 18000 rpm in a 25.50 rotor at 4 ºC. The supernatant was poured into fresh tubes and again centrifuged for 10 min at 18000 rpm in 25.50 rotor at 4ºC. The supernatant was collected and pumped onto two 20 mL DEAE-sepharose fast flow FF columns in tandem, which had been equilibrated in 10 mM Tris/HCl, 1 mM EDTA, pH 8.0 (buffer B). The column was washed with 100 mL buffer B and the protein eluted using a linear gradient from 0-500 mM NaCl in buffer B, gradient volume 500 mL. The absorbance at 214, 256 and 280 nm, and the conductance, were monitored during elution. Samples from selected fractions were analysed by SDS PAGE (10-20% Tris-tricine gels stained with Coomassie; Supporting Figure S21, S22). The fractions with high concentration of S100G-CTDβ1-4 or S100G-CTDβ1-2 were pooled and diluted 3-fold with MilliQ-water and pumped onto 3x5 mL Q sepharose High Performance columns in tandem, pre-equilibrated in 10 mM Tris/HCl, 2.5 mM $CaCl_2$, pH 8.0. The columns were washed with 100 mL 10 mM Tris/HCl, 2.5 mM $CaCl_2$, pH 8.0, and the protein eluted using a linear NaCl gradient from 0-100 mM, followed by a steep gradient from 100 to 500 mM in 10 mM Tris/HCl, 1 mM $CaCl_2$, pH 8.0, total volume 500 mL. The absorbance at 214, 256 and 280 nm, and the conductance, were monitored during elution. Samples from selected fractions were analysed by SDS PAGE (10-20% Tris-tricine gels stained with Coomassie; Supporting Figure S21, S22). Fractions that seemed to contain no other protein than S100G-CTDβ1-2 or S100G-CTDβ1-4 were pooled, concentrated to 10 mL and supplemented with 3 mM EDTA from a concentrated stock before separation by size exclusion chromatography on a Superdex 75 26/600 column in 20 mM sodium phosphate, 0.2 mM EDTA, pH 8.0 (Supporting Figure S21, S22). This last step removes Ca^{2+}, excess EDTA and any remaining contaminants to provide pure material for the kinetics experiments. Samples for CD spectroscopy were prepared by SEC in 20 mM sodium phosphate, pH 8.0. Samples for Ca^{2+}-binding experiments were depleted of Ca^{2+} and EDTA by SEC in pure H_2O on a G25 column (GE healthcare; precleaned with EDTA and then H_2O) and lyophilized.

Expression and purification of Aβ42. Aβ(M1-42), here called Aβ42, was expressed and purified as described [3,4], using isolation of inclusion bodies by sonication, ion exchange in batch mode with isocratic elution using 50 mM NaCl and two SEC steps using column chromatography and stored as multiple identical lyophilized aliquots after monomer isolation. Monomer for the kinetics experiments was isolated from such aliquots, dissolved in 1 mL 6 M GuHCl, pH 8.5, by size exclusion chromatography on a Superdex75 10/300 column in 20 mM sodium phosphate, 0.2 mM EDTA, pH 8.0.

Expression and purification of S100G(E17Q+D19N+E26Q). The S100G mutant E17Q+D19N+E26Q was purified using sonication, heat denaturation of E. coli proteins, anion exchange and size exclusion chromatography as described earlier [5]. Just prior to the kinetics experiment, an aliquot was subjected to SEC on Superdex 75 in 20 mM sodium phosphate, 0.2 mM EDTA, pH 8.0.

Expression and purification of scMn+8 and lysozyme. scMn+8 was expressed and purified as previously described [6]. Chicken egg lysozyme was purchased from Sigma-Aldrich (NR) and purified by passing through an anion exchange resin to remove anionic contaminants. Each protein was subjected to SEC on Superdex 75 in 20 mM sodium phosphate, 0.2 mM EDTA, pH 8.0 to isolate pure monomer in the experimental buffer prior to use in the kinetic assays.

Native mass spectrometry. Lyophilized Aβ42 was dissolved in 200 mM ammonium acetate pH 6.8 for native MS analysis. The buffer of DNJAB6 CTD constructs was exchanged using Micro Bio-Spin spin columns (BioRad) to 200 mM ammonium acetate pH 6.8. Fresh samples were diluted in 200 mM ammonium acetate pH 6.8 to final concentrations of 10-25 µM for MS analysis. Aβ42 and CTD constructs were co-incubated for 10 minutes at 37 ºC prior to native MS analysis.

A Waters Synapt G2S hybrid QToF mass/ion mobility spectrometer equipped with a nano-electrospray source was used for analysis. Samples were injected offline using nano-electrospray by commercial metal coated glass injectors (Thermo Scientific). Ionization was performed in positive ion mode and the instrument parameters were as follows: Capillary voltage 1.5 kV, Sampling cone 50 V, Source offset 50 V, Source temperature 25 °C, Trap voltage 5V, Trap gas 10 mL/min, Helium Gas Flow 100 mL/min, IMS gas flow 50 mL/min, IMS wave velocity 1200 m/s, IMS wave height 40 V. The collisional cross section of the CTD construct was determined from ion mobility drift times by calibration with standard proteins, as has been described earlier [7]. Briefly, experimental drift time values were measured for calibrant proteins of known collisional cross section. Human insulin (Sigma Aldrich) and bovine ubiquitin (Sigma Aldrich) were used as calibrants. The insulin monomer (+3, +4), dimer (+5, +6) and hexamer (+9, +10, +11) ions, and ubiquitin monomer (+4, +5, +6) ions were used for calibration, as they span the m/z range observed for the CTD construct. Reference cross section values were obtained from literature [8].

Chemical crosslinking mass spectrometry. Samples were crosslinked and prepared for mass spectrometric analysis as previously described [9], using the lysine-specific crosslinker BS3 and denaturing electrophoresis from which bands were excised and subjected to in-gel digestion with two proteases, trypsin and chymotrypsin. Mass spectrometric data acquisition and data analysis was done as previously described [9], briefly, the proteolytic digests were subjected to a clean-up step on 3 mm microcolumns and inspected for quality by MALDI mass spectrometry and thereafter peptides were subjected to reversed phase nano-LC-MS/MS on an LTQ-Orbitrap Velos Pro mass spectrometer or on a trapped ion mobility quadrupole time-of-flight mass spectrometer (timsTOF Pro, Bruker Daltonics). After data acquisition the raw files were processed and converted to peak lists (mgf-files) by Mascot Distiller and the software MassAI used for detection and identification of crosslinks with the following settings: trypsin+chymotrypsin, 3 mc, variable mod BS3-H20 and met ox, 3 modif allowed, tolerance 10 ppm MS, 0.1 Da for crosslinked peptides, tolerance 0.2 m/z in MS/MS, 'Also Xlink modified peptides'. The approved MS/MS spectra are provided in the Supporting Information.

Circular dichroism spectroscopy and thermal denaturation. Circular dichroism spectra were recorded between 250 and 185 nm in a 1 mm quartz cuvette using a Jasco J-815 spectropolarimeter equipped with a Peltier element to control the cuvette holder temperature. The scan rate was 10 nm/min, data integration time 8 s, sampling step 1 nm and slit width 2 nm. Three accumulations were averaged for each spectrum and the baseline recorded with buffer in the cuvette subtracted. The stability towards thermal denaturation was monitored by recording the ellipticity at fixed wavelength (222 nm) as a function of temperature from 5 to 95°C using a ramping rate of 1 °C/min, data integration time 32 s and slit width 2 nm. Reverse scans from 95 to 5°C were performed directly after reaching 95°C. The temperature at the denaturation mid-point, T_m, was estimated by fitting eq. 13 to the data:

$$Y = \frac{(k_N T + b_N) + (k_D T + b_D)\exp\left(-\left(\frac{\Delta G^\circ(T)}{RT}\right)\right)}{1 + \exp\left(-\left(\frac{\Delta G^\circ(T)}{RT}\right)\right)} \qquad [15]$$

Where ΔG°(T) is given by the Gibbs-Helmholtz equation:

$$\Delta G°(T) = \Delta H°(T_m)\left(1 - \frac{T}{T_m}\right) + \Delta C_p^o\left(T - T_m - T ln(\frac{T}{T_m})\right) \quad [16]$$

in which $\Delta H°(T_m)$ is the enthalpy change at T_m, and ΔCp the heat capacity change upon denaturation and T the absolute temperature.

Prediction of CD spectra. Theoretical CD spectra were generated of the S100G-CTD constructs using the PDBMD2CD web server (https://pdbmd2cd.cryst.bbk.ac.uk) [10]. The top ranked AlphaFold2 generated structures of S100G-CTDβ1-2, S100G-CTDβ1-4, and S100G-CTDβ1-2Scr were used as inputs for the spectral predictions, and the output CD intensities (in Δε) were multiplied with 3298 to convert into units of mean residue ellipticity (deg cm^2 $dmol^{-1}$).

Aggregation kinetics. Aggregation kinetics experiments were set up in 96-well PEG-ylated polystyrene plates, black with transparent bottom (Corning 3881). A logarithmic dilutions series of each construct in triplicate or quadruplicate in 20 mM sodium phosphate, 0.2 mM EDTA, pH 8.0 was distributed into wells at two times the final concentration, 40 µl per well with the plate at room temperature. Aβ42 monomer was then added using a multi-channel pipette from ice-cold solution in 20 mM sodium phosphate, 0.2 mM EDTA, pH 8.0 at twice the desired final concentration, which was 3 µM Aβ42 and 10 µM ThT, 40 µl per well. The plate was immediately placed in a plate reader (BMG fluostar) at 37°C and the fluorescence emission at 480 nm with 440 nm excitation was monitored through the bottom of the plate.

Seeded aggregation kinetics. Non-seeded aggregation kinetics was setup as above and seeds were collected when reaching the plateau. Seeds and constructs were placed in wells (in triplicate of each condition) at twice the final concentration. Aβ42 monomer was then added using a multi-channel pipette from ice-cold solution in 20 mM sodium phosphate, 0.2 mM EDTA, pH 8.0 at twice the final concentration, which was 3 µM Aβ42 and 10 µM ThT, 40 µl per well. The plate was immediately placed in a plate reader at 37°C and the fluorescence at 480 nm with 440 nm excitation was monitored through the bottom of the plate.

Kinetic analyses. Kinetic analyses of data were performed using the following master equation that describes the time-evolution of the fibril mass concentration, M.

$$\frac{[M]}{[M]_\infty} = 1 - \left(1 - \frac{[M]_0}{[M]_\infty}\right) * \left(\frac{B_+ + C_+}{B_- + C_+} * \frac{B_- + C_+ e^{\kappa t}}{B_+ + C_+ e^{\kappa t}}\right)^{\frac{k_\infty^2}{\bar{k}_\infty \kappa}} e^{-k_\infty t} \quad [1]$$

where the parameters are defined as follows

$$\kappa = \sqrt{2k_+ k_2 [m]_0^{n_2+1}} \quad [2]$$

$$\lambda = \sqrt{2k_+ k_n [m]_0^{n_c}} \quad [3]$$

$$C_\pm = \frac{k_+[P]_0}{\kappa} \pm \frac{k_+[M]_0}{2[m]_0 k_+} \pm \frac{\lambda^2}{(2\kappa^2)} \quad [4]$$

$$k_\infty = \sqrt{\frac{2\kappa^2}{[n_2(n_2+1)} + \frac{2\lambda^2}{n_C}} \quad [5]$$

$$\bar{k}_\infty = \sqrt{k_\infty^2 - 4C_+ C_- \kappa^2} \quad [6]$$

$$B_{\pm} = \frac{k_{\infty} \pm \bar{k}_{\infty}}{2\kappa} \qquad [7]$$

In these relations, $[m]_0$ is the initial monomer concentration, $[P]_0$ is aggregate number concentration at the start of the reaction, $[P]_\infty$ is the aggregate number concentration after reaction completion, $[M]_0$ is the mass concentration of fibrils at the start of the reaction and $[M]_\infty$ is the mass concentration of fibrils at the end. k_n, k_2, k_+ are the rate constants for primary nucleation, secondary nucleation and elongation respectively. n_c and n_2 are the monomer scalings of primary and secondary nucleation, respectively. The values
n_c = 2, and n_2 = 2 were used for fitting of all aggregation kinetics based on previous results for Aβ42 [3].

Normalization of ThT fluorescence and curve-fitting were performed using the Amylofit online software [11]. For each tested construct, data for all replicates at the same concentration were included in the analysis.

Cryo-TEM. Specimens for cryo-TEM were prepared in an automatic plunge freezer system (Leica EM GP). The climate chamber temperature was kept at 21 °C, and relative humidity was ≥ 90 % to minimize loss of solution during sample preparation. The specimens were prepared by placing 4 µL solution on glow discharged lacey formvar carbon coated copper grids (Ted Pella) and blotted with filter paper before being plunged into liquid ethane at -180 °C. This leads to vitrified specimens, avoiding component segmentation and rearrangement, and the formation of water crystals, thereby preserving original microstructures. The vitrified specimens were stored under liquid nitrogen until measured. A Fischione Model 2550 cryo transfer tomography holder was used to transfer the specimen into the electron microscope, JEM 2200FS, equipped with an in-column energy filter (Omega), which allows zero-loss imaging. The acceleration voltage was 200 kV and zero-loss images were recorded digitally with a TVIPS F416 camera using SerialEM under low dose conditions with a 10 eV energy selecting slit in place.

Ca^{2+} binding. The macroscopic Ca^{2+}-binding constants, K_1 and K_2, for S100G-CTDβ1-4 and S100G- CTDβ1-2 were determined from competitive Ca^{2+} titrations versus the chromophoric Ca^{2+} chelator Quin2 in 2 mM Tris/HCl, pH 7.5. The buffer was prepared in a plastic container, using de-calcified pH electrode and stored with 10 mL Chelex-100 resin in a dialysis bag (3.5 kDa cutoff, three-times boiled before use) in the container. The total Ca^{2+} concentration of the buffer was determined to be 0.3 µM based on the Quin2 absorbance at 263 nm in the absence and presence of excess EDTA and in the presence excess Ca^{2+}[12]. In the competitive titrations, the total quin2 concentration was 26.6 µM (determined from the absorbance of Ca^{2+}-loaded Quin2 at 239.5 nm using $\varepsilon_{239.5} = 4.2 \cdot 10^4\ M^{-1}cm^{-1}$) and the protein concentration was around 30 µM based on weight. $CaCl_2$ was added stepwise from a 2.5 mM stock and the absorbance at 263 nm, A, was recorded at each titration step after 45 s equilibration. Equation 8 was fitted to the data

$$A = (A_{QCa} - A_Q)\frac{[Ca^{2+}]}{[Ca^{2+}] + K_{DQ}} + A_Q \qquad [8]$$

where K_{DQ} is the equilibrium dissociation constant of the quin 2-Ca^{2+} complex, A_Q the absorbance of free quin2 and A_{QCa} the absorbance of the quin 2-Ca^{2+} complex and $[Ca^{2+}]$ the free Ca^{2+} concentration. At each titration point $[Ca^{2+}]$ was solved from the total Ca^{2+} concentration, C_{Ca}, using the Newton-Raphson algorithm, from the following relation between total, C_{Ca}, and free Ca^{2+} concentration:

$$C_{Ca} = C_Q\frac{[Ca^{2+}]}{[Ca^{2+}] + K_{DQ}} + C_P\frac{K_1[Ca^{2+}] + 2K_1K_2[Ca^{2+}]^2}{1 + K_1[Ca^{2+}] + K_1K_2[Ca^{2+}]^2} + [Ca^{2+}] \qquad [9]$$

The first term in equation [9] models the single-site binding to quin2 and the second term models the binding of two ions to the protein by the Adair equation [13], in which K_1 is the equilibrium association constant for the first bound Ca^{2+} and K_2 for the last bound Ca^{2+}. The geometric mean

of K_1 and K_2 corresponds to the average Ca^{2+} affinity of binding two Ca^{2+} ions to each S100G construct,

$$K_{av} = \sqrt{K_1 K_2} \qquad [10]$$

whereas the ratio of K_2 to K_1 provides a measure of the cooperativity of Ca^{2+} binding through the relation

$$\Delta\Delta G_{\eta=1} = -RTln\left(\frac{4K_2}{K_1}\right) \qquad [11]$$

If the two sites have equal affinity, then

$$\Delta\Delta G = \Delta\Delta G_{\eta=1} \qquad [12]$$

and positive cooperativity is at hand if $\Delta\Delta G < 0$.

Surface plasmon resonance (SPR). The SPR experiments were performed using a Biacore 3000 instrument (GE Healthcare), with CM3 sensors with carboxylated dextran surface. The flow rate was 10 µL/min throughout using 20 mM phosphate, 0.2 mM EDTA, pH 8.0 with 0.005% Tween20 as the flow buffer. The central fraction of Aβ42 monomer from gel filtration in 20 mM phosphate, 0.2 mM EDTA, pH 8.0 was incubated for 2 h at 37°C to form fibrils under quiescent conditions, followed by two minutes tip sonication to produce short (ca. 50 nm) fibrils for immobilization. The carboxylic acid groups on the CM3 sensor surface were activated with a mixture of EDC and NHS to enable standard amine coupling chemistry. The short fibrils were diluted 10-fold into 10 mM sodium acetate buffer at pH 3.0 and injected over the activated surface, after which remaining sensor surface groups were inactivated by injection of 50 µl 1 M ethanolamine in water. Fibrils were then grown on the chip using multiple injections of Aβ42 monomer. Each construct was injected over the immobilized fibrils for 10 or 20 minutes, followed by buffer flow.

The dissociation phase data were fitted with a single exponential decay with rate constant k_{off}, plus a constant slope with rate constant k describing dissociation of monomers from fibril ends.

$$y = A\, e^{(-k_{off}t)} + B - k\, t \qquad [13]$$

The association phase data were fitted with the following equation with the effective rate constant equal to $c\, k_{on} + k_{off}$, where c is the injected construct concentration:

$$y = A\,(1 - e^{(-(c\, k_{on} + k_{off})t)}) + B \qquad [14]$$

Native mass spectrometry to investigate the DNAJB6 constructs

All six constructs were monitored for their interaction with Aβ using native MS. We have previously applied native MS to investigate the interaction between the DNAJB6 chaperone and Aβ [14]. We then obtained evidence for interactions between DNAJB6 and Aβ oligomers which appear to bind strongly to DNAJB6. This is consistent with the observation that DNAJB6 inhibits primary nucleation and prevent amyloid fibril formation of aggregation-prone peptides at remarkably low sub-stoichiometric molar ratios of chaperone to peptide [15,16]. In this work we used native MS to investigate the DNAJB6 constructs, and their possible interaction with Aβ.

Data for CTD are presented in Figure S3, with mass spectra of CTD and CTD STA under native conditions indicating that both these constructs are folded and relatively pure. The molecular weight agrees with theoretical MW calculated from the amino acid sequences. Whereas dimers of CTD are observed, this is not the case for CTD STA.

Supporting Figures (S1-S22)

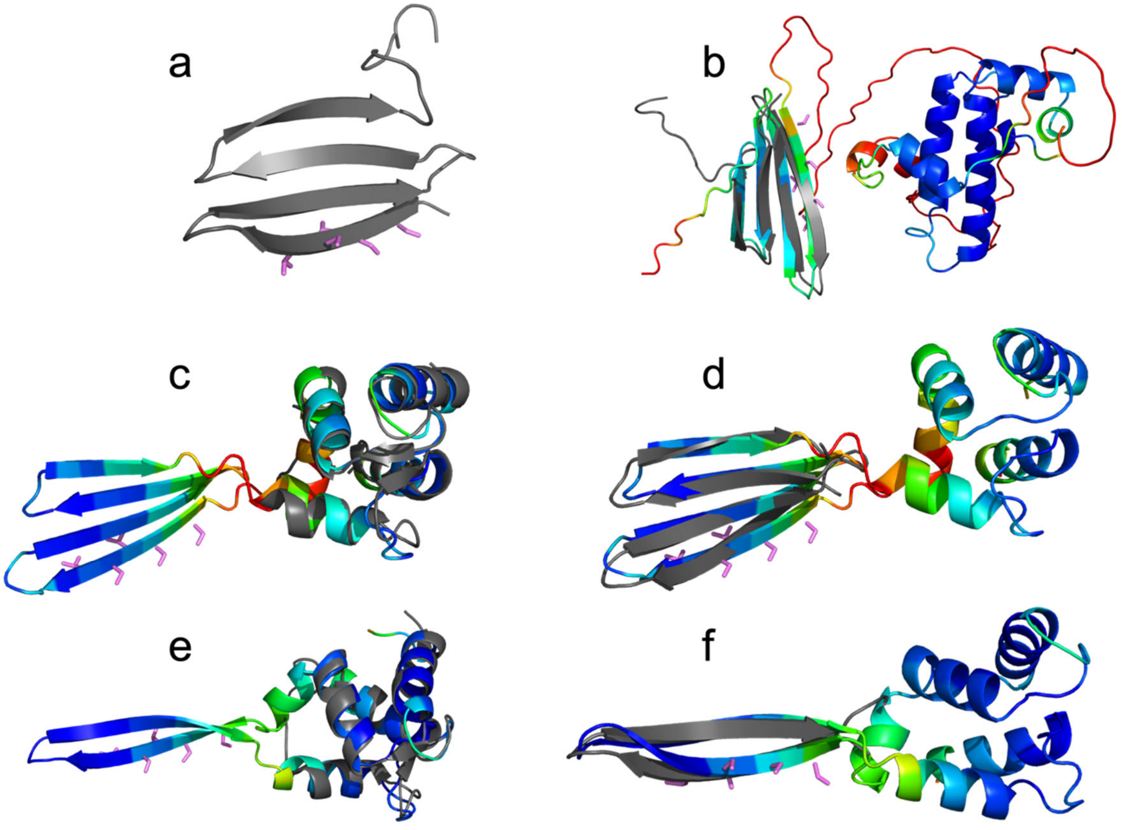

g. CTD

MNFKSISTSTKMVNGRKITTKRIVENGQERVEVEEDGQLKSLTINGKEQLLRLDNK

h. CTD STA

MNFKAIAAAAKMVNGRKITTKRIVENGQERVEVEEDGQLKSLTINGKEQLLRLDNK

i. S100G-CTDβ1-2

MKSPEELKRIFEKYAAKEGDPDQLSKDELKLLIQAEFPSLLKGMNFKSISTSTKMVNGR
KITTKRIVENGSTLDDLFQELDKDGDGEVSFEEFQVLVKKISQ

j. S100G-CTDβ1-2STA

MKSPEELKRIFEKYAAKEGDPDQLSKDELKLLIQAEFPSLLKGMNFKAIAAAAKMVNGR
KITTKRIVENGSTLDDLFQELDKDGDGEVSFEEFQVLVKKISQ

k. S100G-CTDβ1-4

MKSPEELKRIFEKYAAKEGDPDQLSKDELKLLIQAEFPSLLKGMGGGNFKSISTSTKMV
NGRKITTKRIVENGQERVEVEEDGQLKSLTINGGGSTLDDLFQELDKDGDGEVSFEEFQ
VLVKKISQ

l. S100G-CTDβ1-4STA

MKSPEELKRIFEKYAAKEGDPDQLSKDELKLLIQAEFPSLLKGMGGGNFKAIAAAAKMV
NGRKITTKRIVENGQERVEVEEDGQLKSLTINGGGSTLDDLFQELDKDGDGEVSFEEFQ
VLVKKISQ

m. S100G-CTDβ1-2Scr

MKSPEELKRIFEKYAAKEGDPDQLSKDELKLLIQAEFPSLLKGMKITVSVNRSTFGKTS
TMEIKGNNRIKSTLDDLFQELDKDGDGEVSFEEFQVLVKKISQ

Fig. S1. **Structure prediction and amino acid sequences of the studied DNAJB6b constructs**. **a-f)** Structural models predicted using the online version of AlphaFold2 w/ MMseqs2, and no templates where used. [2,17]. Side chains of mutated residues are showed in pink in all figures **a-f**. In figure **b-f**, the residue color represents the local confidence of the prediction by AlphaFold2 (the exception is the side chains of the mutated residues in DNAJB6b, which are showed in pink for the predictions). Color code from low confidence to high: red-yellow-green-cyan-blue (further description below). **a)** The C-terminal domain (CTD) of DNAJB6b, solved structure using NMR, PDB-entry 7jsq. **b)** DNAJB6b structure prediction from AlphaFold, aligned with CTD from **a**, in grey. **c)** The prediction of S100G with an insert of the CTD of DNAJB6b, but without the last 10 residues that can be seen as an unstructured tail to the beta-sheets in **a** (S100G-CTDβ1-4 for short). The prediction is aligned with an NMR-solved structure of S100G, PDB-entry 2bcb (grey). **d)** The same prediction as in **c**, aligned with the solved structure of DNAJB6b CTD from **a** (grey), but without the last 10 residues to match the insert sequence. **e)** and **f)** Similar as **c** and **d**, but with only the sequence of DNAJB6b that build up the beta-sheet pair containing the mutated residues (called S100G-CTDβ1-2). **g-m)** Amino acid sequences of CTD, CTD STA, S100G-CTDβ1-2, S100G-CTDβ1-2STA, S100G-CTDβ1-4, S100G-CTDβ1-4STA and S100G-CTDβ1-2Scr.

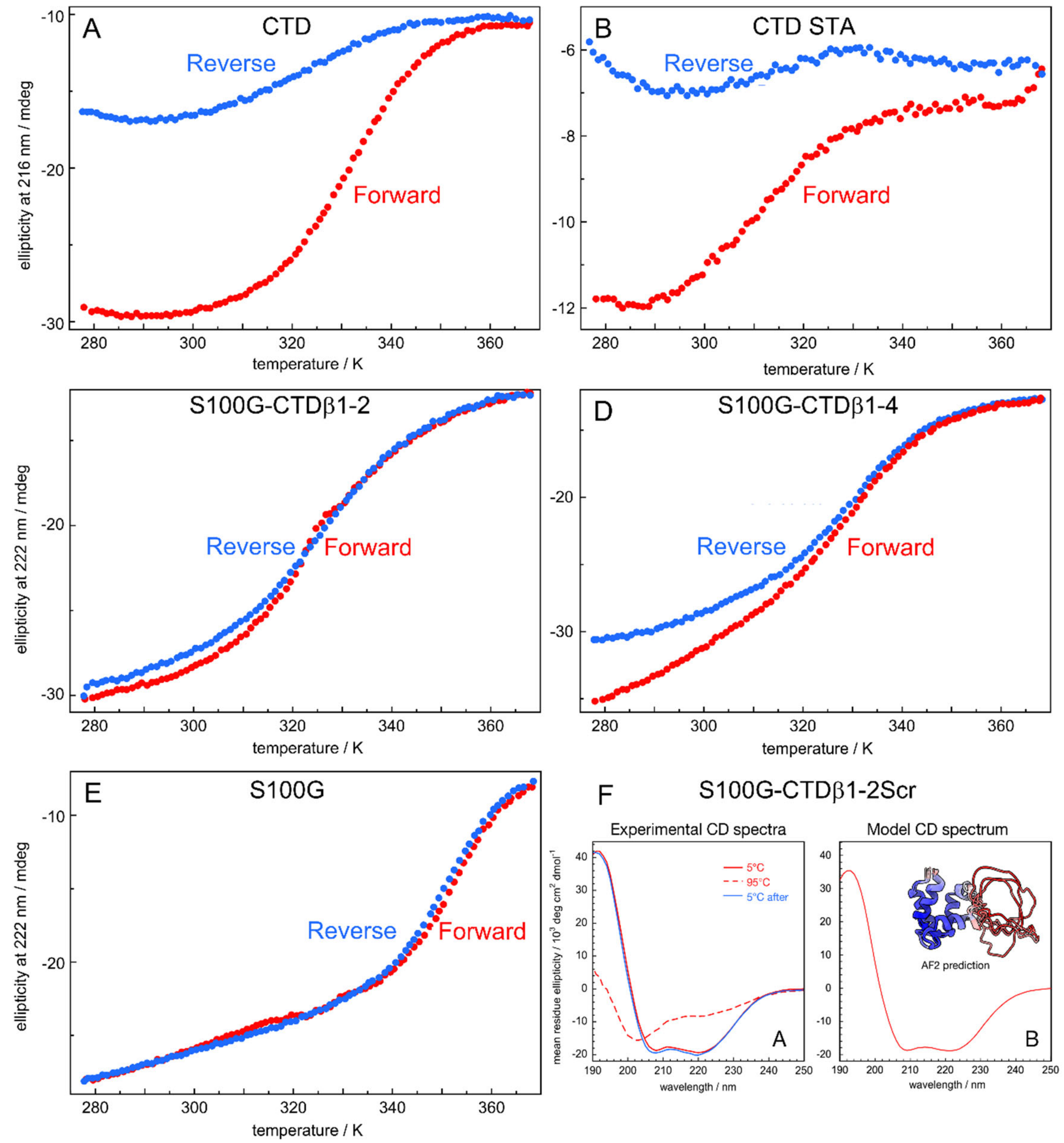

Fig. S2. Figure S2. Thermal stability and reversibility of CTD, CTD STA, S100G-CTDβ1-2, S100G-CTDβ1-4 and S100G. The ellipticity versus temperature for the forward thermal scan from 5 °C to 95 °C (red) and the reverse scan back to 5 °C (blue) measured at 216 nm for CTD (**A**) and CTD STA (**B**) and at 222 nm for S100G--CTDβ1-2 (**C**), S100G--CTDβ1-4 (**D**) and S100G (**E**). Panel **F** shows the CD spectra of S100G-CTDβ1-2Scr at 5 °C before (red) and after (blue) a thermal scan, and at 95 °C (red dashed line). All proteins were studied in 20 mM sodium phosphate with 200 μM EDTA, because S100G does not denature below 100 °C in the presence of Ca^{2+}. The denaturation of S100G and S100G-CTDβ1-2Scr is fully reversible, of S100G--CTDβ1-2 almost fully reversible and of S100G-CTDβ1-4 approximately 80% reversible, indicating that in this case only the S100G scaffold refolds upon cooling. The denaturation of CTD and especially CTD STA is the least reversible of the proteins studied.

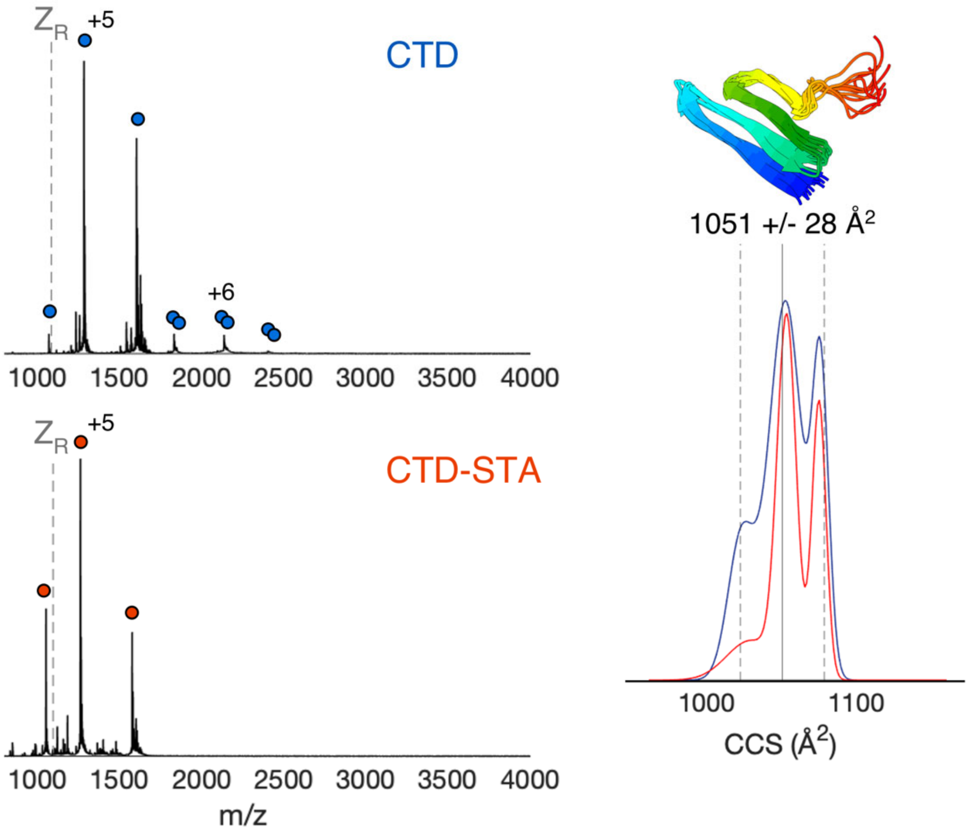

Figure S3. Native MS of CTD and CTD STA. Mass spectra recorded of CTD and CTD STA under native conditions (200 mM ammonium acetate pH 6.8) (left) with the Rayleigh limit indicated (Z_R). Ion mobility profile for the +5 ions (right) of CTD (blue) and CTD STA (red). The calculated collisional cross section (CCS) of the 7jsq.pdb NMR ensemble (gray, calculated using IMPACT ([18])) is shown together with the experimental CCS profiles.

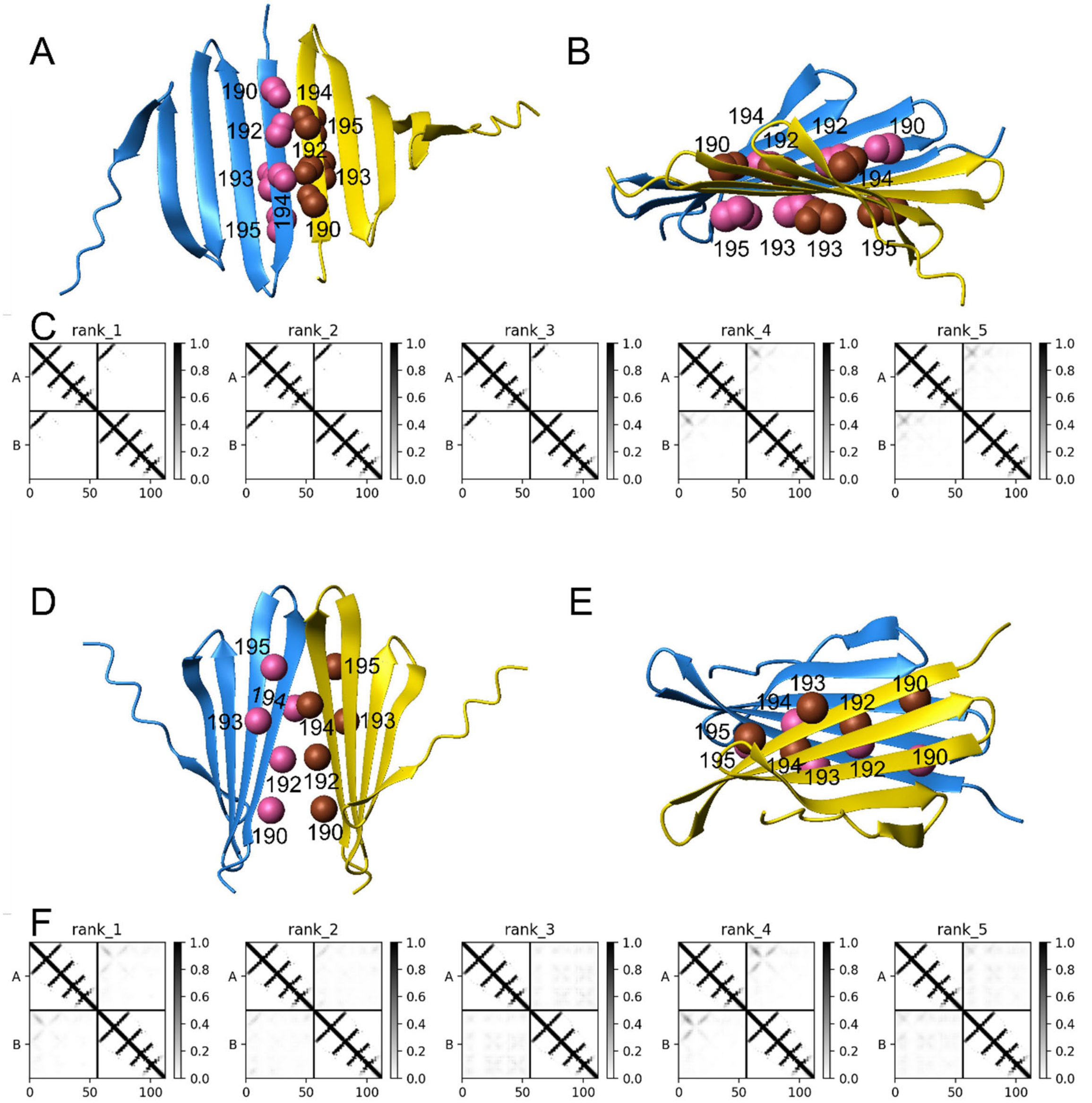

Figure S4. Alphafold2 predictions of dimeric CTD and dimeric CTD STA. A,B) Alphafold2 prediction of possible CTD dimer, with dark blue indicating the more confidently predicted elements. The five mutated serine and threonine residues S190, S192, T193, S194, are indicated by their residue number. **C)** contact diagrams for five predictions. **D,E**) Alphafold2 prediction of possible CTD STA dimer, with dark blue indicating the more confidently predicted elements. **F)** contact diagrams for five predictions of CTD STA dimer.

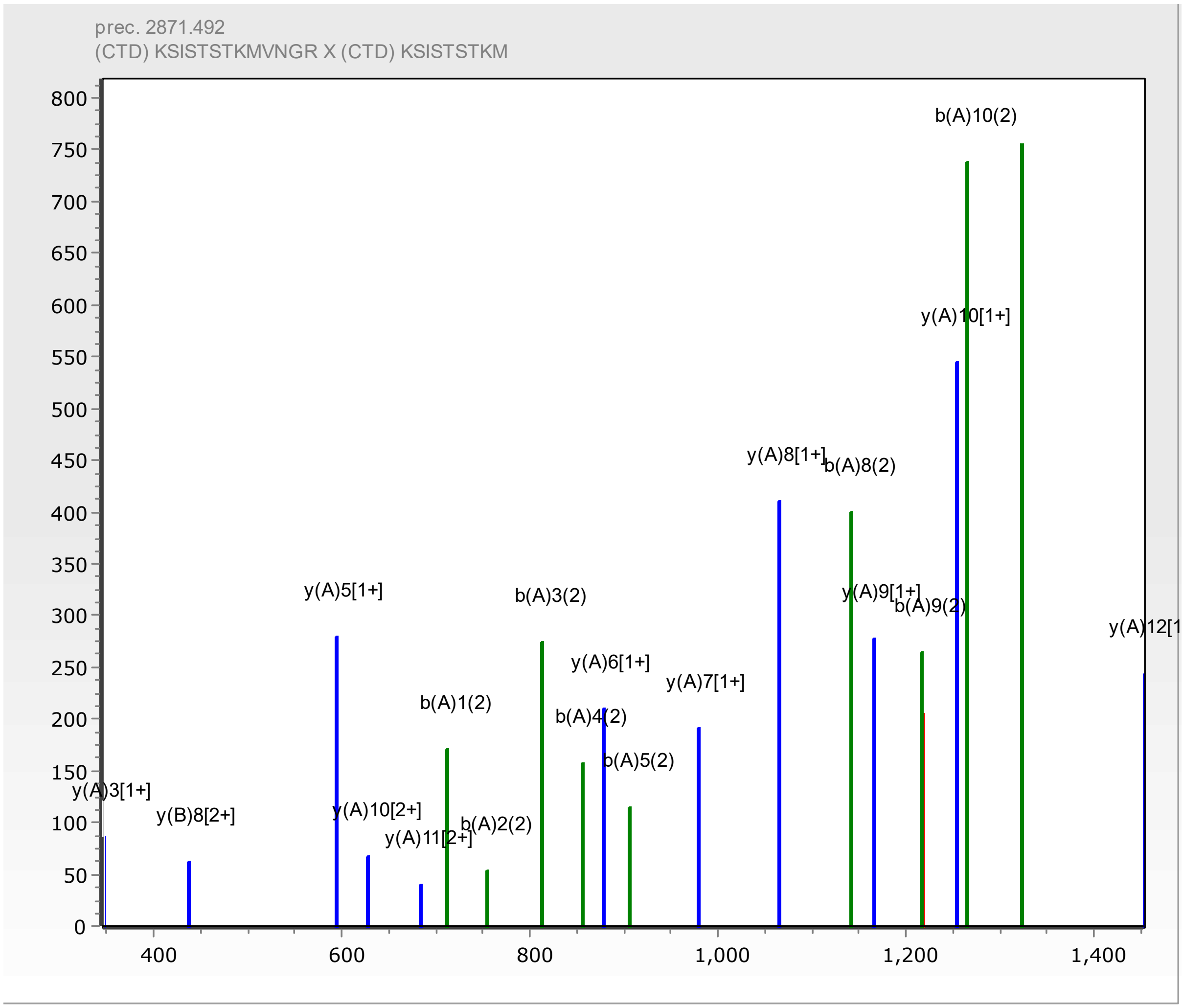

Figure S5A. MSMS spectrum of crosslinked CTD dimer. Detected K189-K189 crosslink in the band corresponding to CTD dimer (Table 1, line 1).

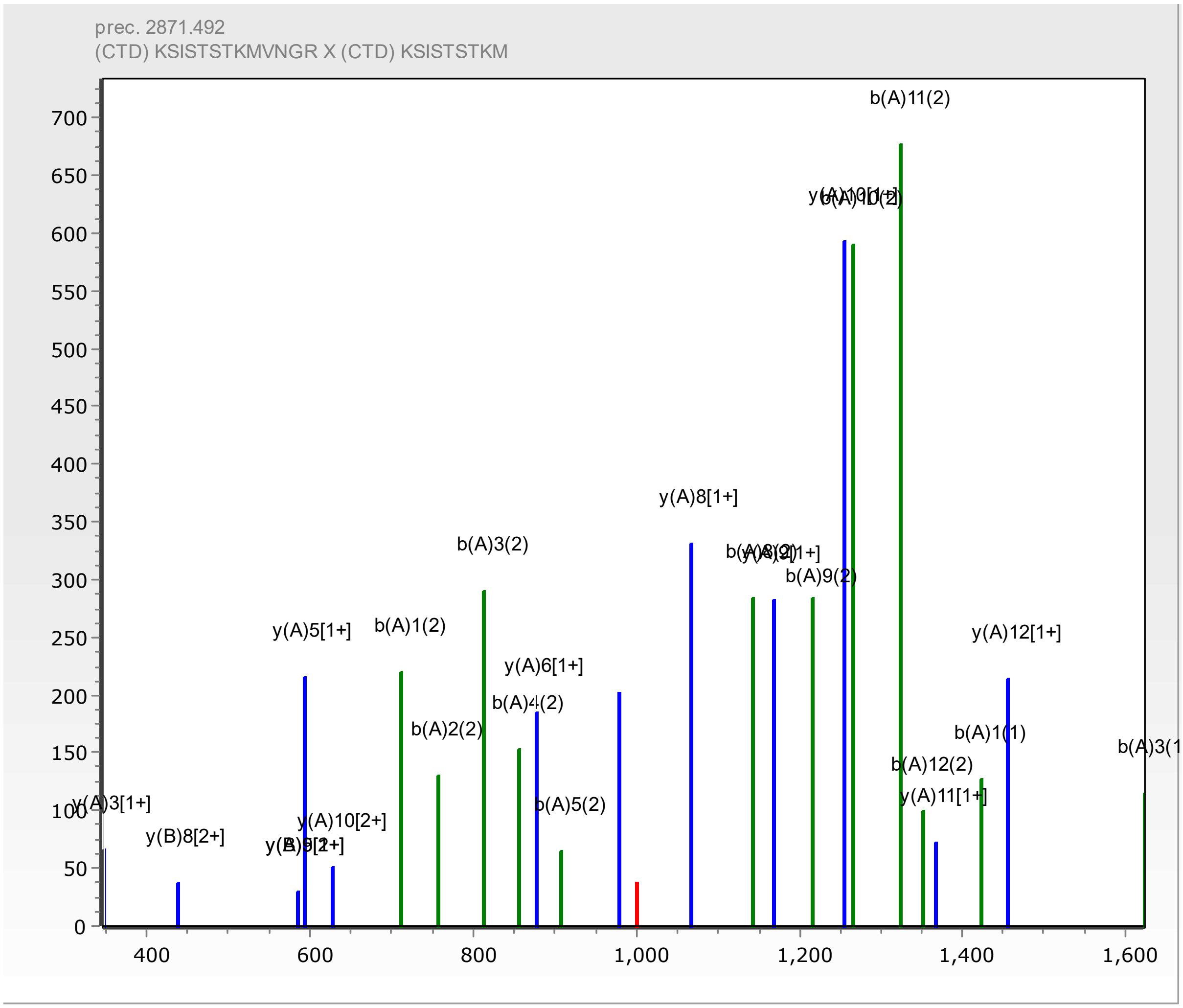

Figure S5B. MSMS spectrum of crosslinked CTD tetramer. Detected K189-K189 crosslink in the band corresponding to CTD tetramer (Table 1, line 2).

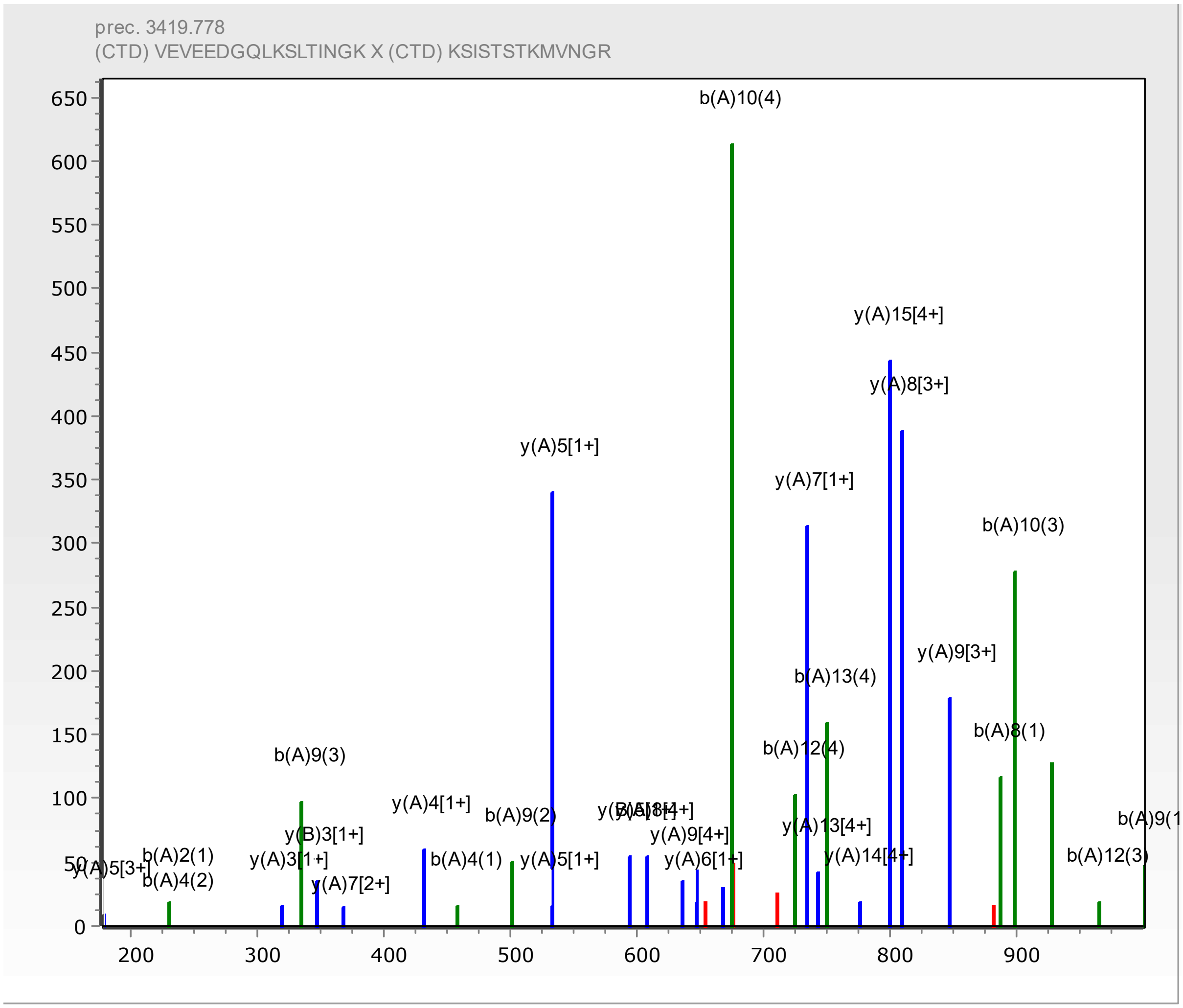

Figure S5C. MSMS spectrum of crosslinked CTD tetramer. Detected K189-K225 crosslink in the band corresponding to CTD tetramer (Table 1, line 3).

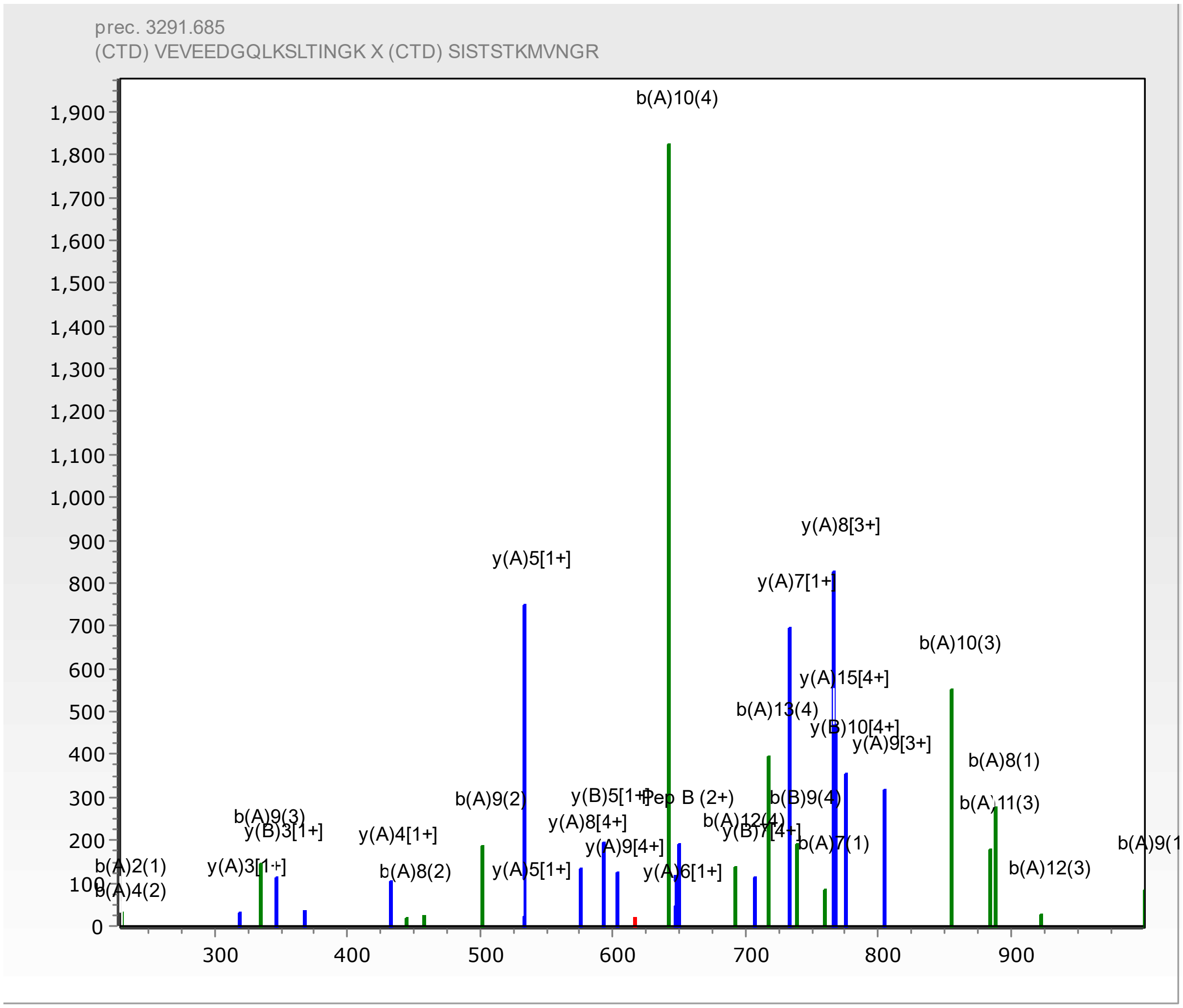

Figure S5D. MSMS spectrum of crosslinked CTD tetramer. Detected K196-K225 crosslink in the band corresponding to CTD tetramer (Table 1, line 4).

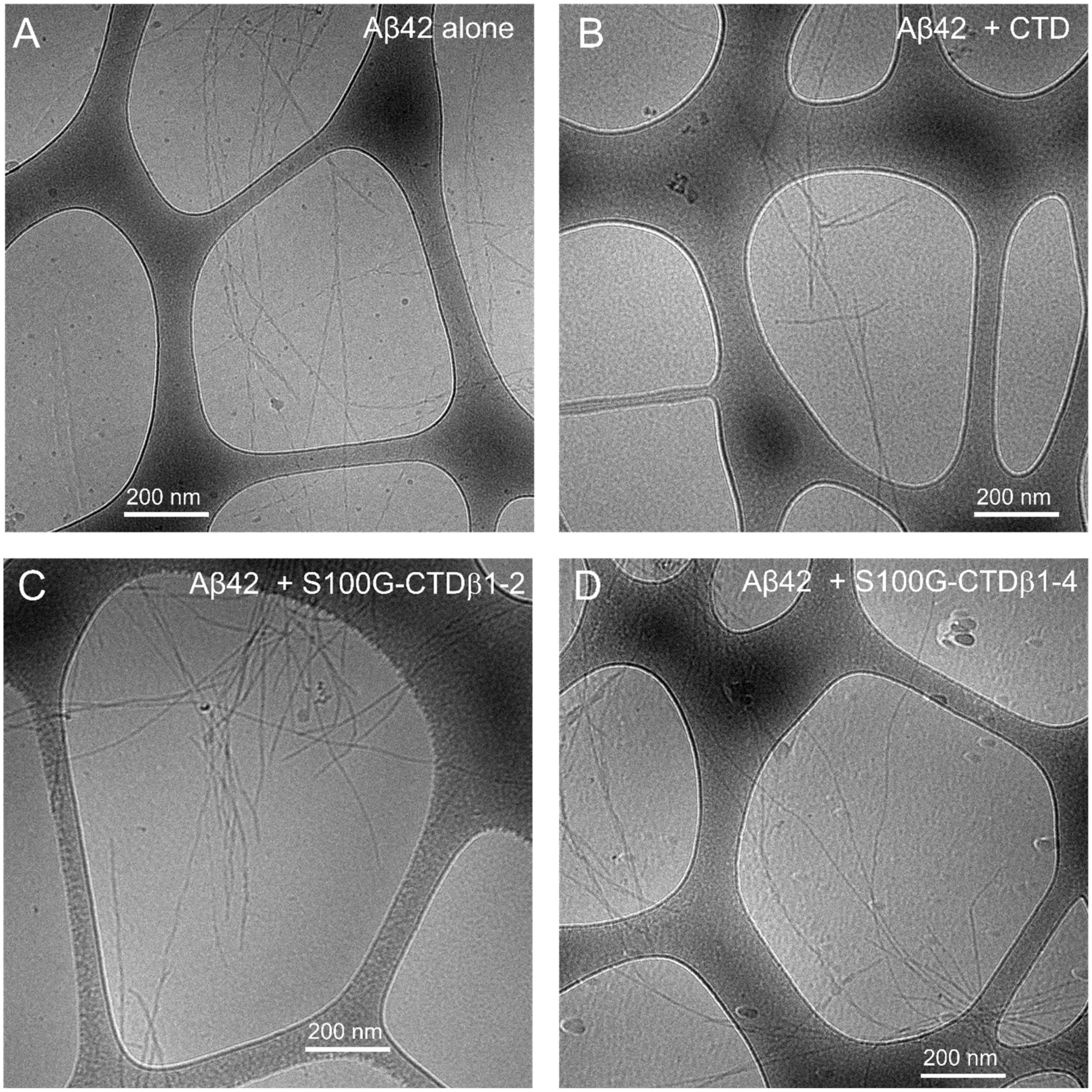

Figure S6. Cryo-EM images of fibrils at the end of the aggregation reaction in 20 mM sodium phosphate, 0.2 mM EDTA, 0.02% NaN_3, pH 8.0, formed from **A**) Aβ42 alone, **B**) Aβ42 plus CTD, **C**) Aβ42 plus S100G-CTDβ1-2, **D**) Aβ42 plus S100G-CTDβ1-4. Fibrils are formed in presence of all three DNAJB6 constructs but fewer fibrils display a distinct twist with separation between the two filaments compared to fibrils formed from Aβ42 alone. Scale bar = 200 nm.

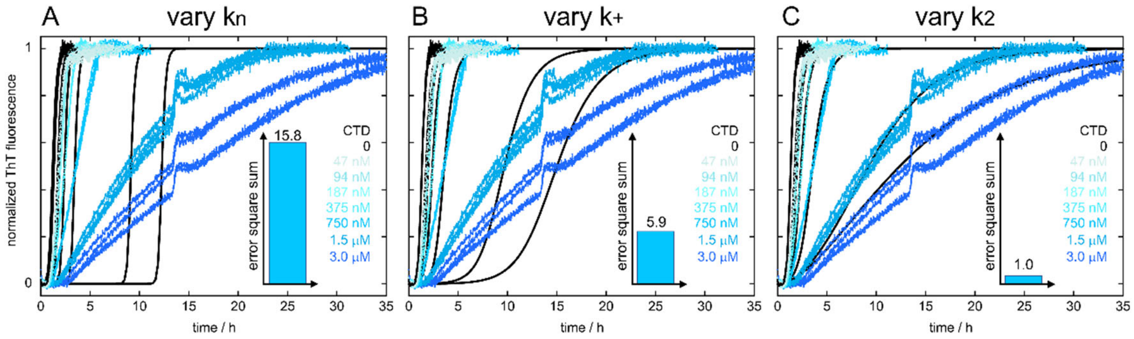

Figure S7. Kinetic analysis of Aβ42 aggregaion in the presence of CTD. Curve-fitting to data for Aβ42 aggregation in the presence of CTD, residues 187-241 of DNAJB6. The aggregation kinetics were recorded starting from Aβ42 monomer in the absence and presence of CTD at concentrations ranging from 47 nM to 3 μM. The color scheme is shown in each panel. The same data is shown three times and the fits allow a single rate constant at a time to be curve specific: **A**) k_n, **B**) k_+, **C**) k_2. The inset in each panel shows the error sqaure sum for each fit relative to the best one, i.e. relatve to the one allowing k_2 to be curve specific. The data shown in this figure are the same data as shown in Figure 4A, and are here shown together with alternative curve fitting where different parameters are alowed to vary.

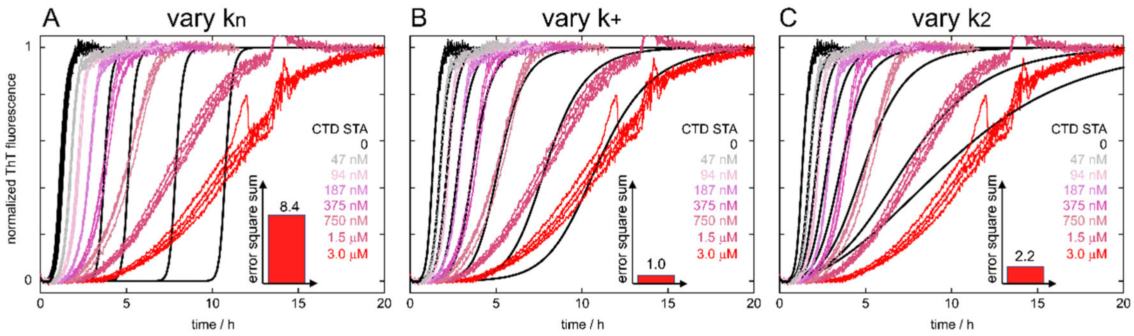

Figure S8. Kinetic analysis of Aβ42 aggregaion in the presence of CTD STA. Curve-fitting to adata for Aβ42 aggregation in the presence of CTD STA, residues 187-241 of DNAJB6 with 5 S/T o A substitutions. The aggregation kinetics were recorded starting from Aβ42 monomer in the absence and presence of CTD STA at concentrations ranging from 47 nM to 3 μM. The color scheme is shown in each panel. The same data is shown three times and the fits allow a single rate constant at a time to be curve specific: **A**) k_n, **B**) k_+, **C**) k_2. The inset in each panel shows the error sqaure sum for each fit relative to the best one, i.e. relatve to the one allowing k_2 to be curve specific. The data shown in this figure are the same data as shown in Figure 4B, and are here shown together with alternative curve fitting where different parameters are alowed to vary.

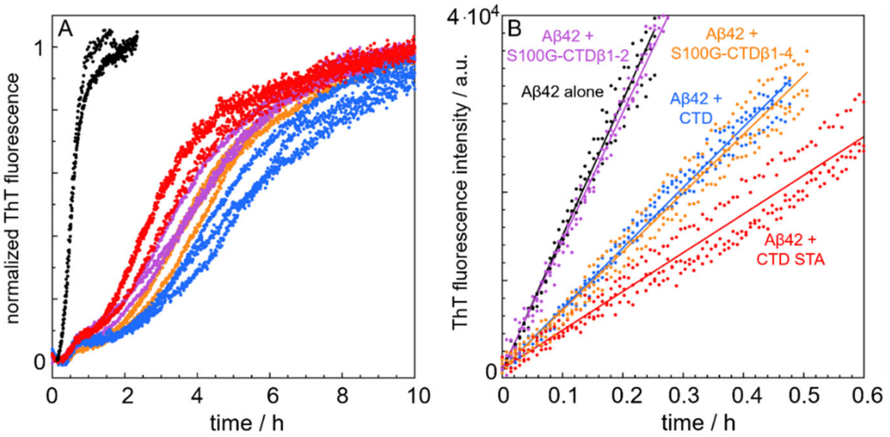

Fig S9. Seeded aggregation kinetics. A) Normalized ThT fluorescence intensity for reactions started with 1% seed and 3 μM Aβ42 monomer and none (black) or 3 μM of S100G-CTDβ1-4 (orange), S100G-CTDβ1-2 (purple), CTD (blue) or CTD STA (red). Tree replicates are shown in each case. **B) I**nitial ThT fluorescence intensity as a function of time for reactions started with 50% seed and 3 μM Aβ42 monomer and none (black) or 3 μM of S100G-CTDβ1-4 (orange), S100G-CTDβ1-2 (purple), CTD (blue) or CTD STA (red). Three replicates are shown in each case and the solid lines show linear fits to the combined data from three replicates.

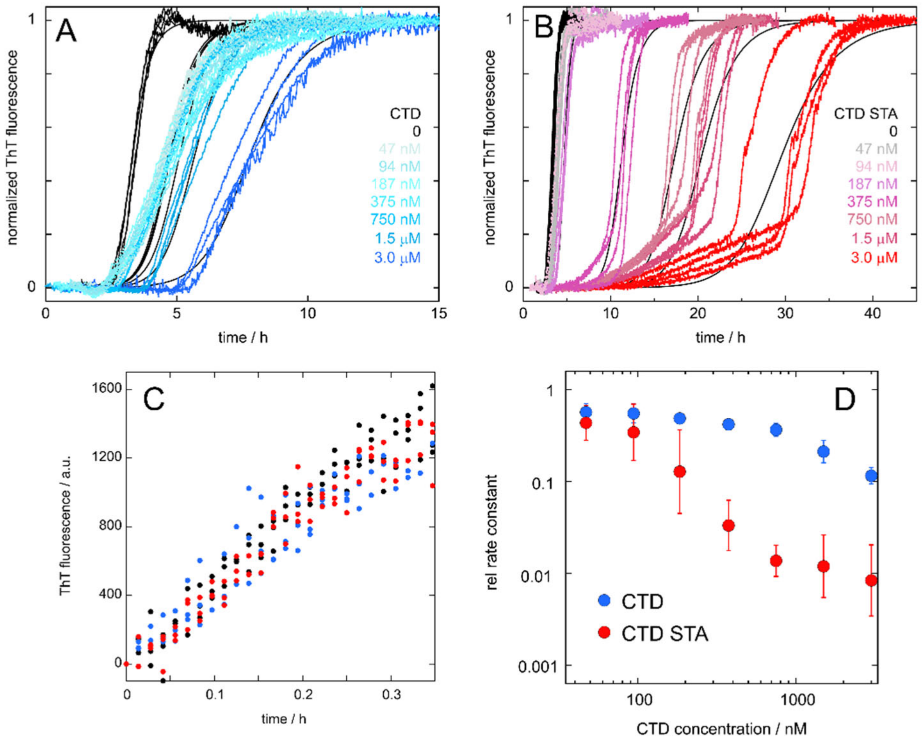

Figure S10. A Aβ42 aggregation in the presence of CTD pr CTD STA at 15ºC. Kinetic analyses of non-seeded data for 3 μM Aβ42 in the presence of CTD (A) or CTD STA (B), using k_2 as the only fitted parameter. Data from heavily seeded experiments are shown in Panel C for 3 μM Aβ42 in the absence (black) and presence of 3 μM CTD (blue) or 3 μM CTD STA (red. The effects on k_2 are shown in panel D as averages and standard deviation over 12 replicates at each inhibitor concentration.

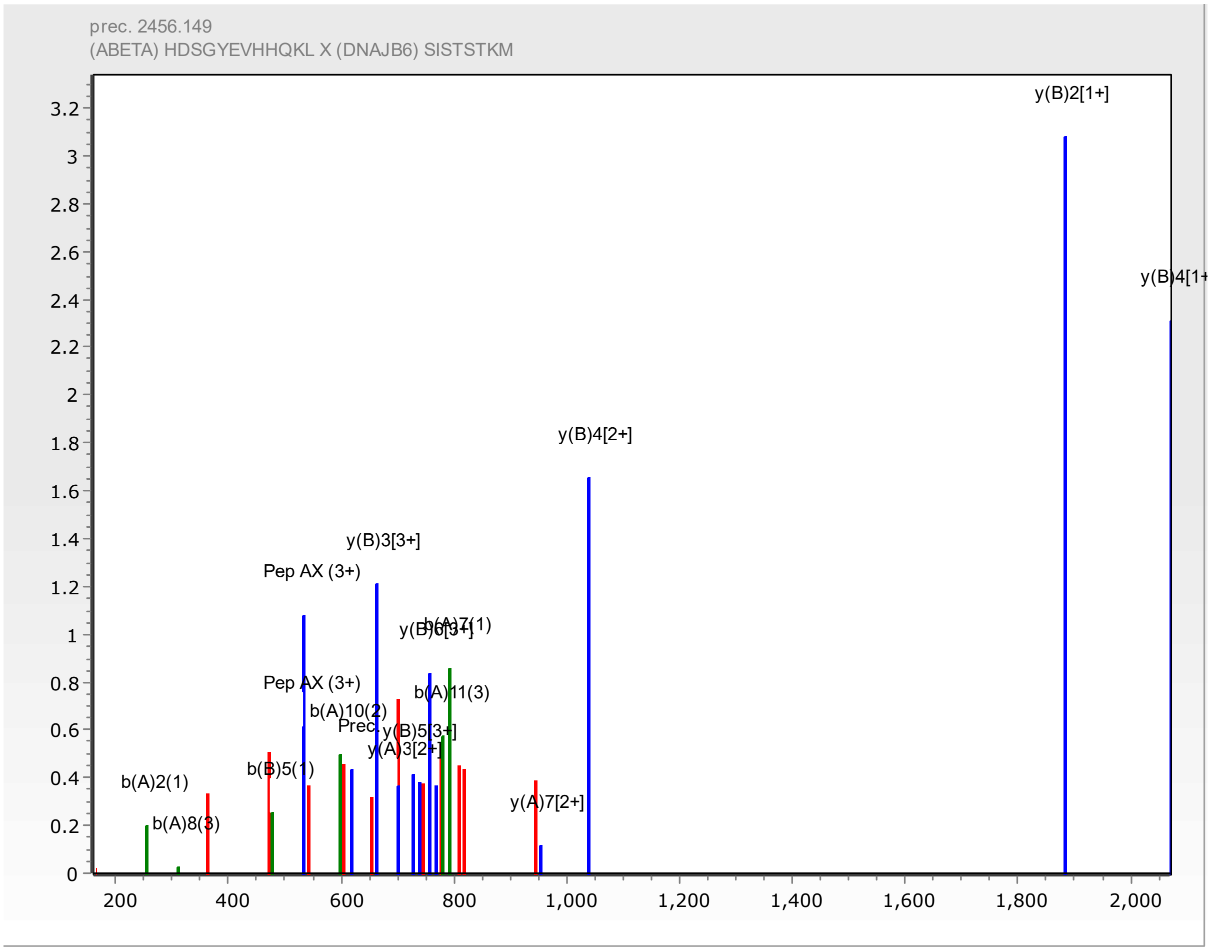

Figure S11A. MSMS spectrum of crosslinked Aβ42-DNAJB6 complex. Detected K16-K196 crosslink (Table S2, line 1).

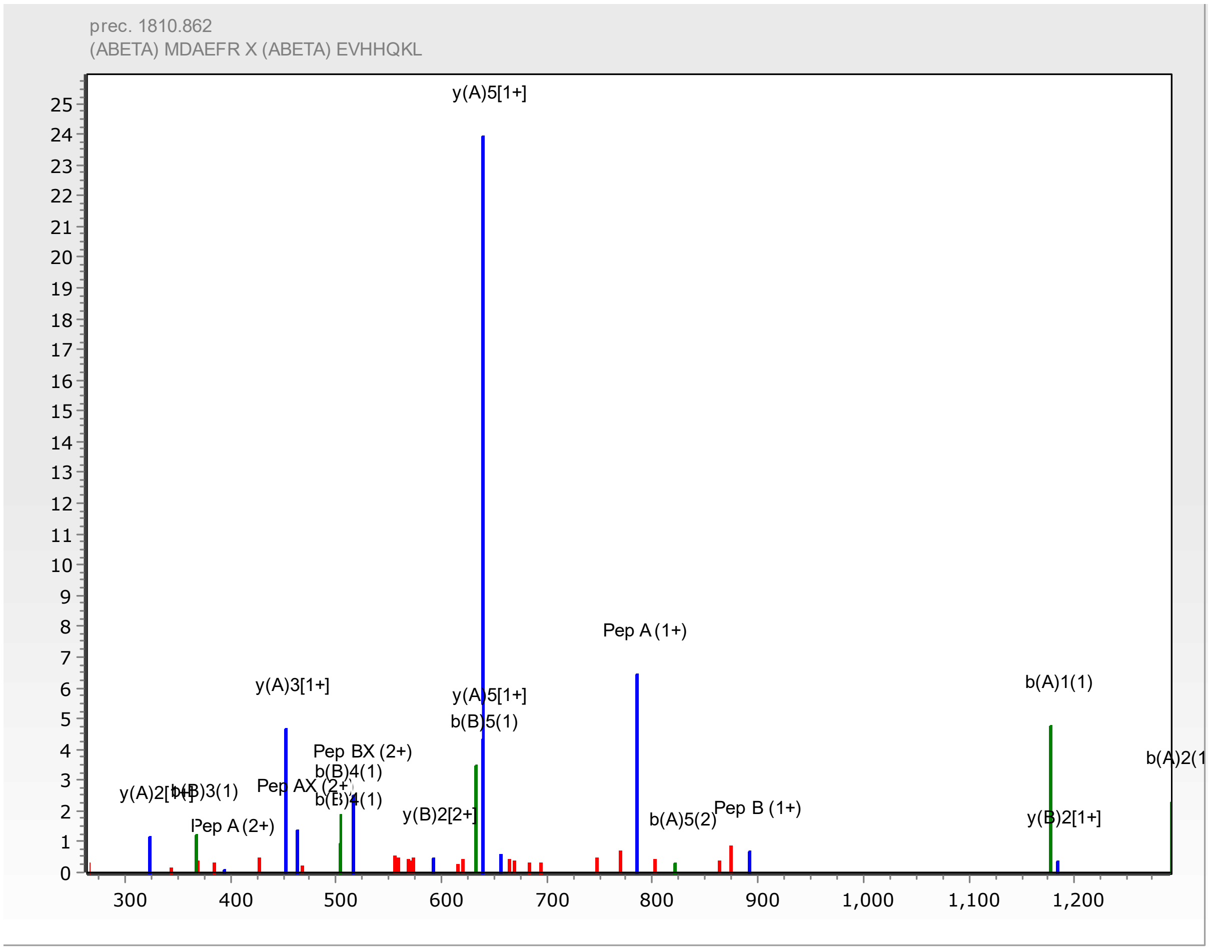

Figure S11B. MSMS spectrum of and internal Aβ42- Aβ42 crosslink found in the Aβ42-DNAJB6 complex. Detected N-term to K16 crosslink (Table S2, line 2).

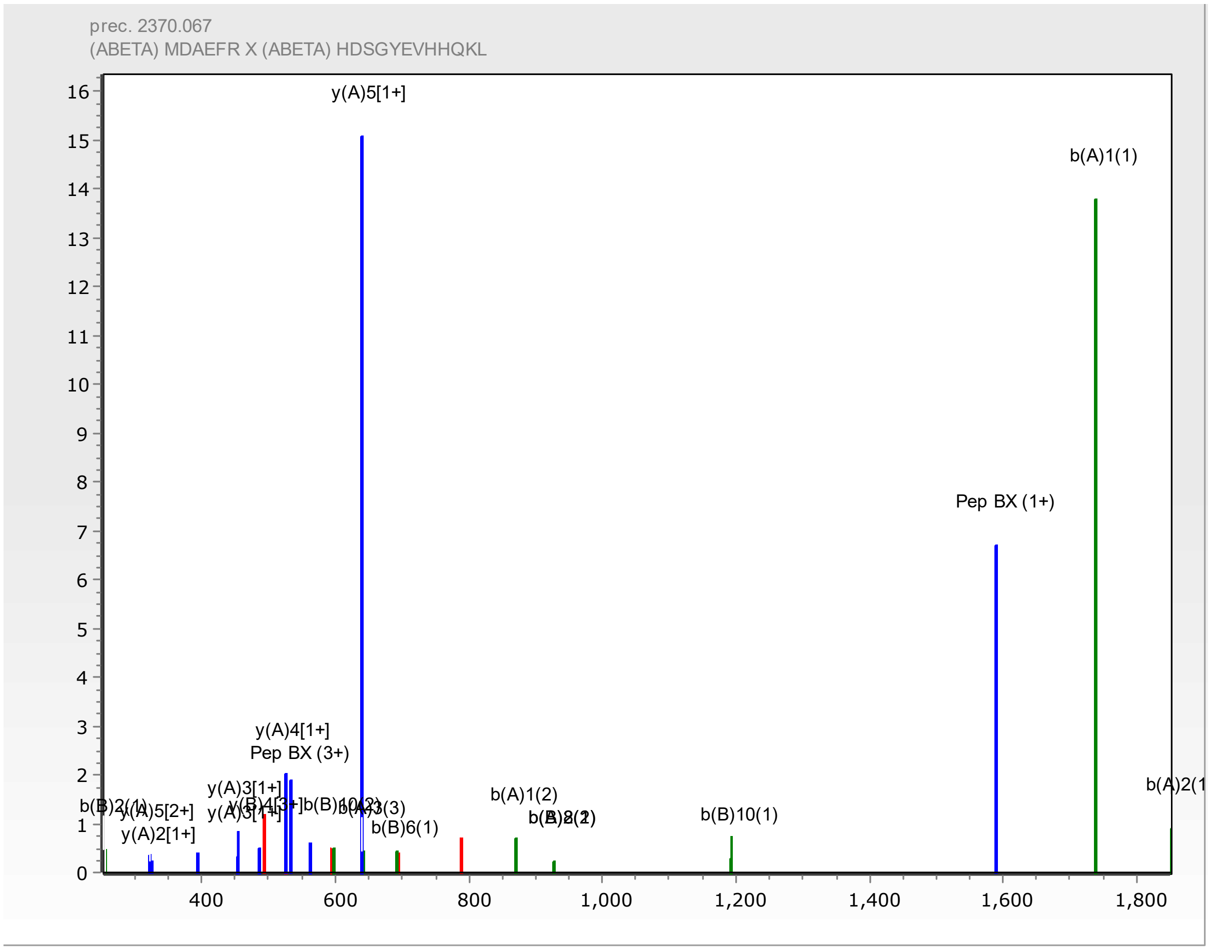

Figure S11C. MSMS spectrum of and internal Aβ42-Aβ42 crosslink found in the Aβ42-CTD complex. Detected N-term to K16 crosslink (Table S2, line 4).

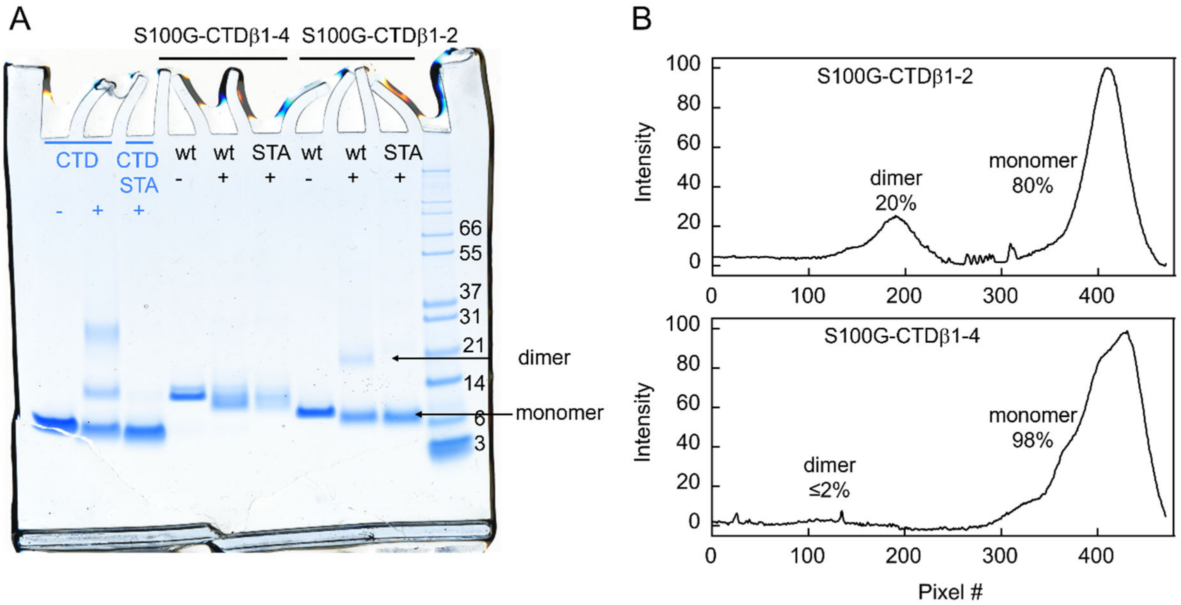

Figure S12A,B. Crosslinking of S100G-CTD grafts. A) SDS-PAGE analysis of S100G-CTDβ1-2, S100G-CTDβ1-2STA, S100G-CTDβ1-4 and S100G-CTDβ1-4STA with (+) and without (-) crosslinking prior to analysis. The first three gel lanes, marked blue, are the same as shown in Figure 2. **B)** Image J analysis of the lanes after cross-linking, with the intensity calculated as 250-gray value, normalized from 0 to 100, and the percentage of each species deduced from peak integration indicated under the respective peak. Based on this distribution and the total construct concentration 10 μM, the concentration of each species was calculated assuming equal staining per monomer in each species.

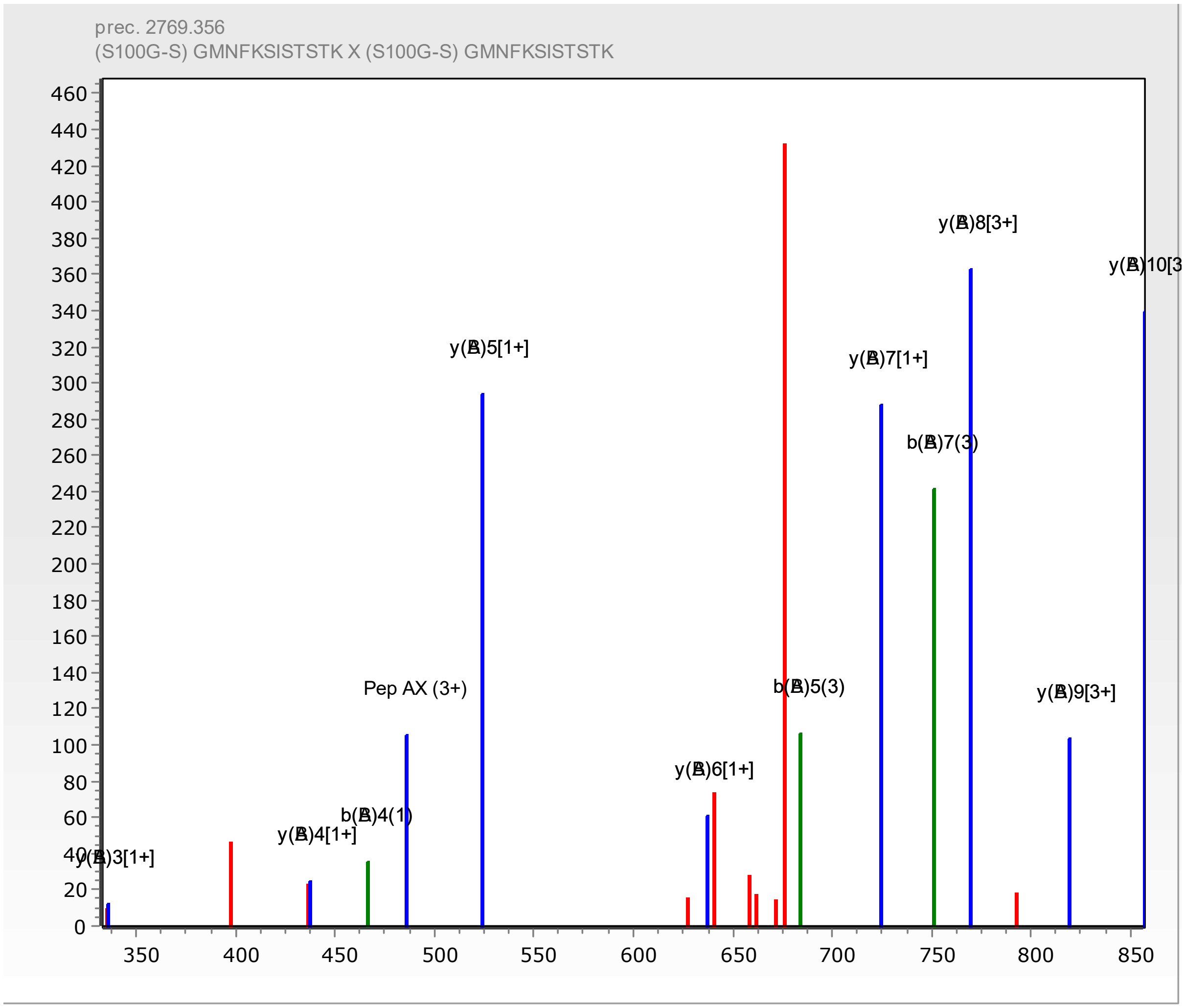

	Precursor mass	Charge	Score	Crosslinked peptides	Lys A*	Lys B*
S100G-CTDβ1-2 dimer	693.347	4+	30.8	GMNFKSISTSTK + GMNFKSISTSTK	K189	K189

Figure S12C. MSMS-spectrum for crosslinked S100G-CTDβ1-2 dimer. This spectrum supports the identification of a K189-K189 crosslink in the S100G-CTDβ1-2 dimer.

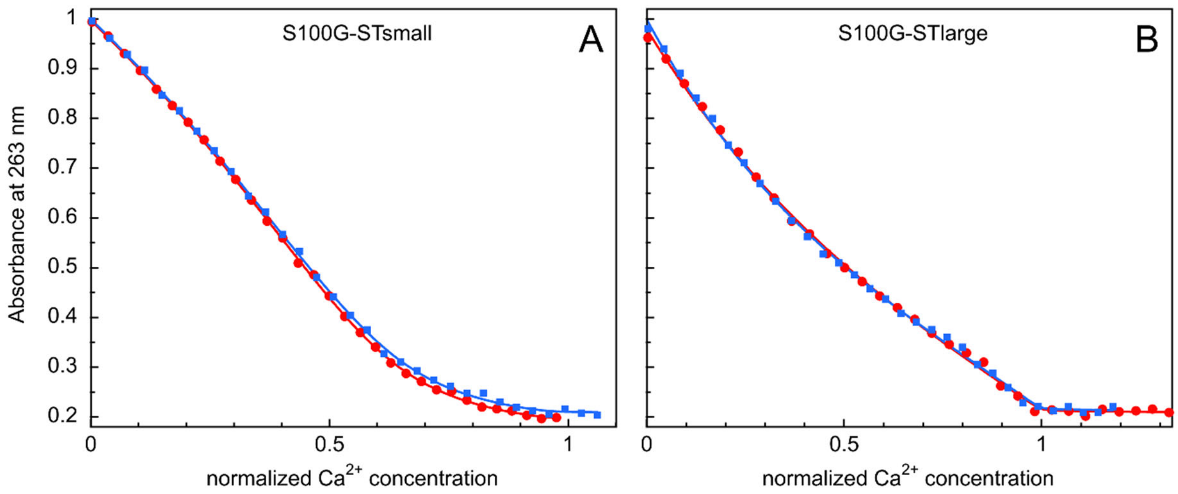

Figure S13. Ca^{2+} binding. Normalized quin2 absorbance at 263 nm *versus* total Ca^{2+} concentration. The Ca^{2+} concentration is normalized such that 1.0 corresponds to the sum of the quin2 concentration plus 2 times the protein concentration. Two repeats of each experiment are shown with red circles and blue squares, respectively. The filled signals are data points and the solid lines are fits of equation 8 to the data.

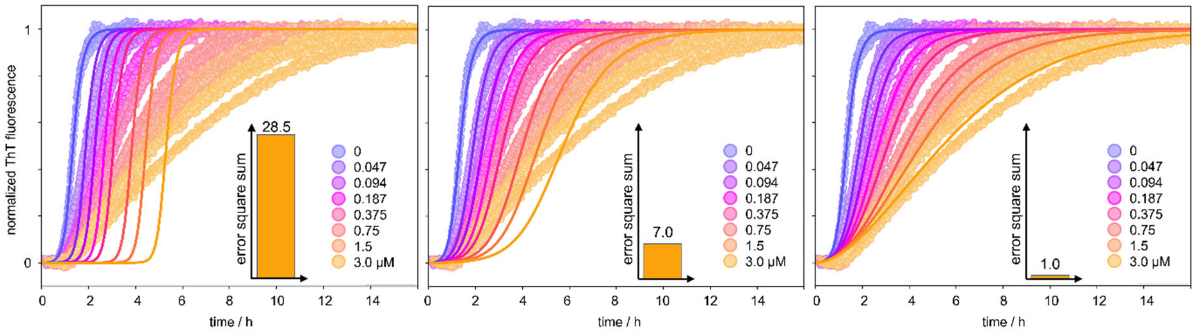

Figure S14. Kinetic analysis of Aβ42 aggregation in the presence of S100G-CTDβ1-2. Curve-fitting to data for Aβ42 aggregation in the presence of S100G-CTDβ1-2, residues 187-212 of DNAJB6 grafted between the two EF-hands of S100G. The aggregation kinetics were recorded starting from 3 μM Aβ42 monomer in the absence and presence of S100G-CTDβ1-2 at concentrations ranging from 47 nM to 3 μM. The color scheme is shown in each panel. The same data is shown three times and the fits allow a single rate constant at a time to be curve specific: **A**) k_n, **B**) k_+, **C**) k_2. The inset in each panel shows the error sqaure sum for each fit relative to the best one, i.e. relatve to the one allowing k_2 to be curve specific.

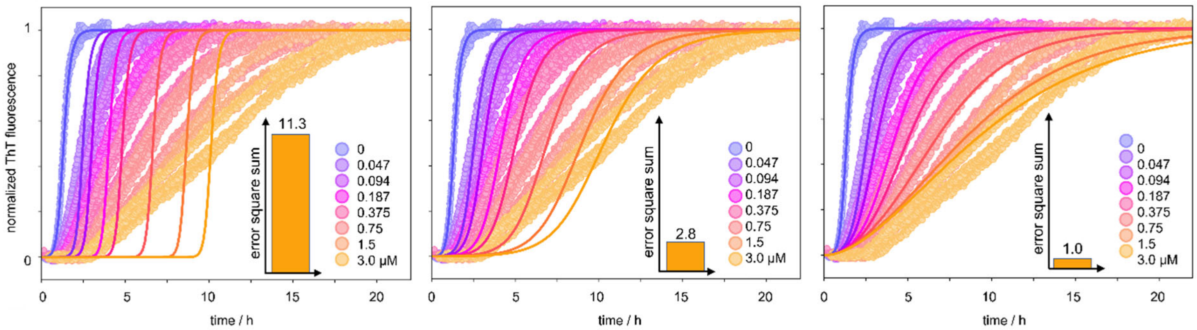

Figure S15. Kinetic analysis of Aβ42 aggregation in the presence of S100G-CTDβ1-4. Curve-fitting to data for Aβ42 aggregation in the presence of S100G-CTDβ1-4, residues 187-231 of DNAJB6 grafted between the two EF-hands of S100G. The aggregation kinetics were recorded starting from 3 μM Aβ42 monomer in the absence and presence of S100G-CTDβ1-4 at concentrations ranging from 47 nM to 3 μM. The color scheme is shown in each panel. The same data is shown three times and the fits allow a single rate constant at a time to be curve specific: **A**) k_n, **B**) k_+, **C**) k_2. The inset in each panel shows the error sqaure sum for each fit relative to the best one, i.e. relatve to the one allowing k_2 to be curve specific.

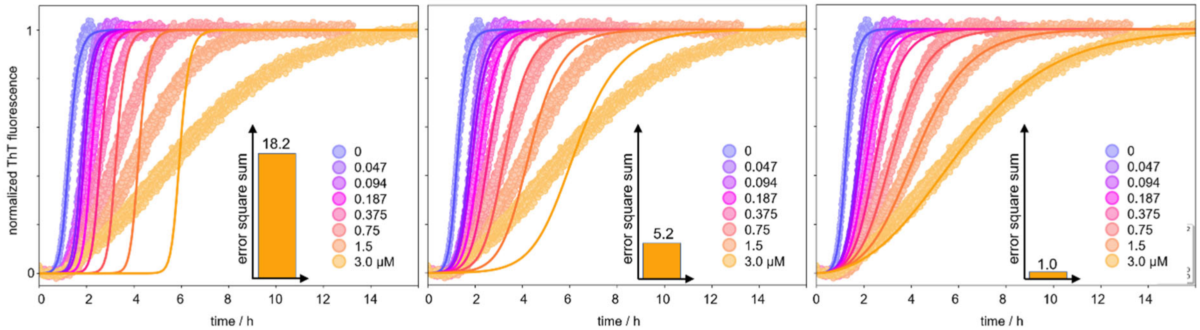

Figure S16. Kinetic analysis of Aβ42 aggregation in the presence of S100G-CTDβ1-2STA. Curve-fitting to aggregat data for Aβ42 aggregation in the presence of S100G-CTDβ1-2STA. The aggregation kinetics were recorded starting from 3 μM Aβ42 monomer in the absence and presence of S100G-CTDβ1-2STA at concentrations ranging from 47 nM to 3 μM. The color scheme is shown in each panel. The same data is shown three times and the fits allow a single rate constant at a time to be curve specific: **A**) k_n, **B**) k_+, **C**) k_2. The inset in each panel shows the error sqaure sum for each fit relative to the best one, i.e. relatve to the one allowing k_2 to be curve specific.

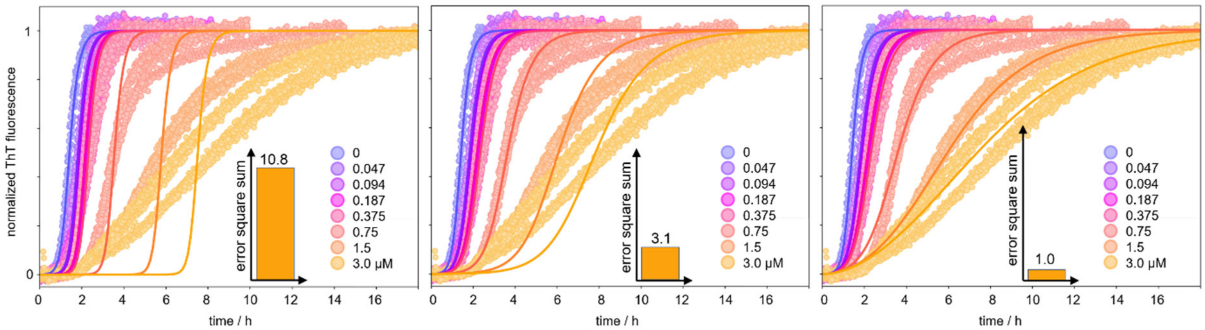

Figure S17. Kinetic analysis of Aβ42 aggregation in the presence of S100G-CTDβ1-4STA. Curve-fitting to aggregat data for Aβ42 aggregation in the presence of S100G-CTDβ1-4STA. The aggregation kinetics were recorded starting from 3 μM Aβ42 monomer in the absence and presence of S100G-CTDβ1-4STA at concentrations ranging from 47 nM to 3 μM. The color scheme is shown in each panel. The same data is shown three times and the fits allow a single rate constant at a time to be curve specific: **A**) k_n, **B**) k_+, **C**) k_2. The inset in each panel shows the error sqaure sum for each fit relative to the best one, i.e. relatve to the one allowing k_2 to be curve specific.

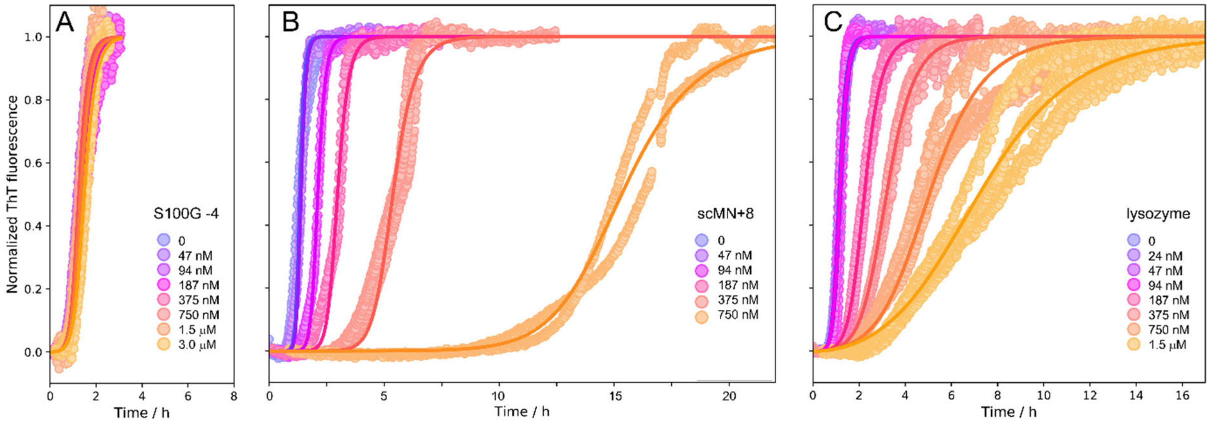

Figure S18. Aβ42 aggregation in the presence of S100G-4, scMN+8 and lysozyme. The aggregation kinetics were recorded starting from 3 μM Aβ42 monomer in the absence and presence of S100G -4 (A), scMn+8 (B) or lysozyme (C) at concentrations ranging from 24 nM to 3 μM as indicated by the color scheme in each panel. The fitted curves allowed k_2 to vary ber concentration, while k_n and k_+ were fitted globally.

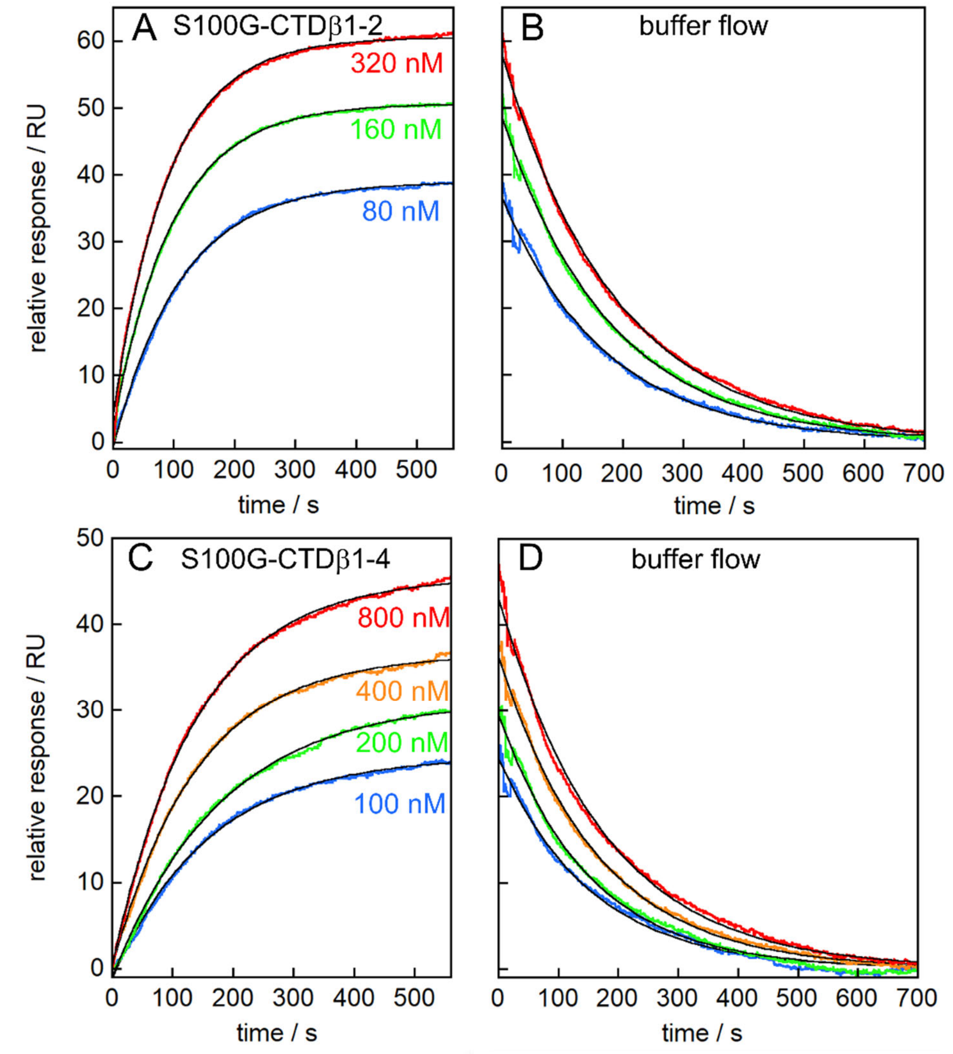

Figure S19. SPR data for S100G-CTDβ1-2 and S100G- CTDβ1-4. Sensorgrams recorded during the for the injection of S100G-CTDβ1-2 (A) or S100G-CTDβ1-4 (C) over immobilized Aβ42 fibrils at 80-800 nM shown in different colours as indicated in each panel. The sensorgrams recorded under the following buffer flow are shown in panel B and D. All data are shown with colors and the fitted curves are shown in black.

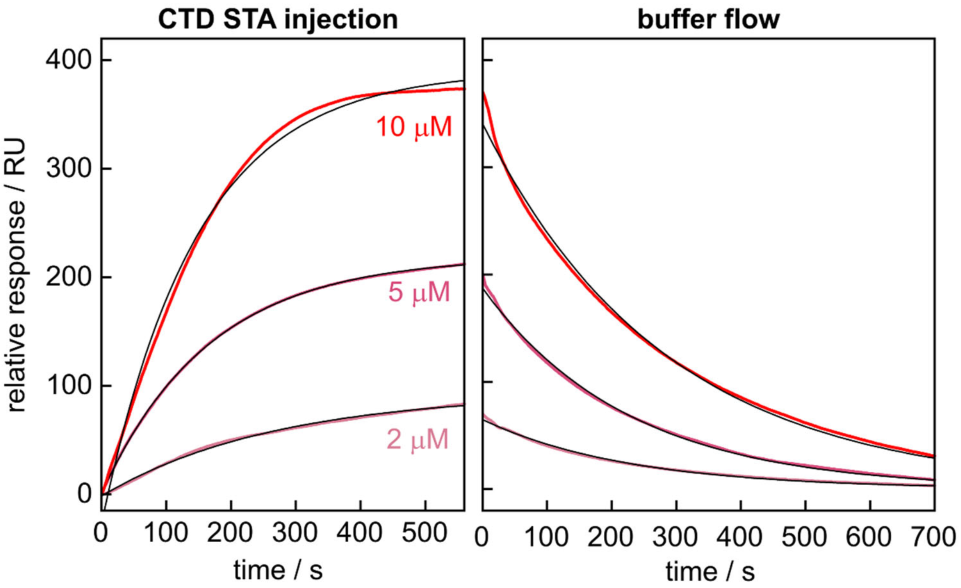

Figure S20. SPR data in 20 mM sodium phosphate, 0.2 mM EDTA, pH 8.0, 0.005% Tween 20, for the interaction of CTD STA with immobilized Aβ42 fibrils at 2 (light pink), 5 (pink) and 10 (red) μM CTD STA (left), and the data obtained under buffer flow following the respective CTD injection (right). All data are shown with colors and the fitted curves are shown in black.

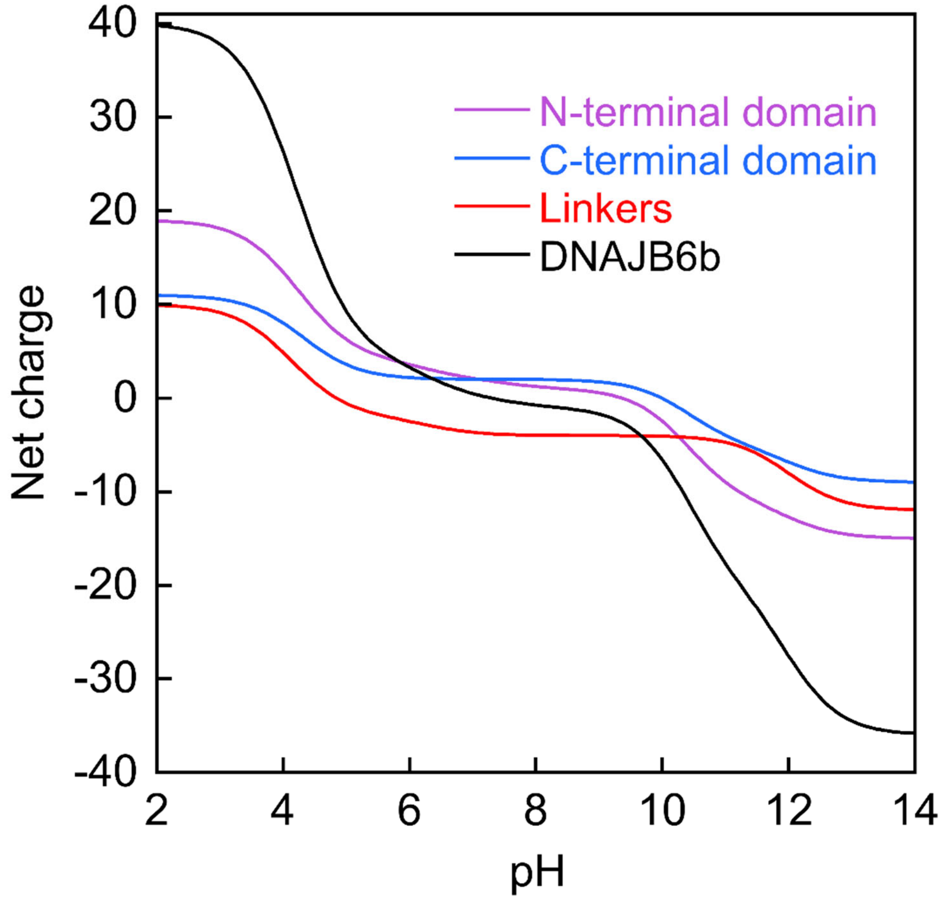

Figure S21. pH dependence of DNAJB6 net charge. The net charge versus pH was calculated assuming model compaound values for all ionizable sidechains [19]

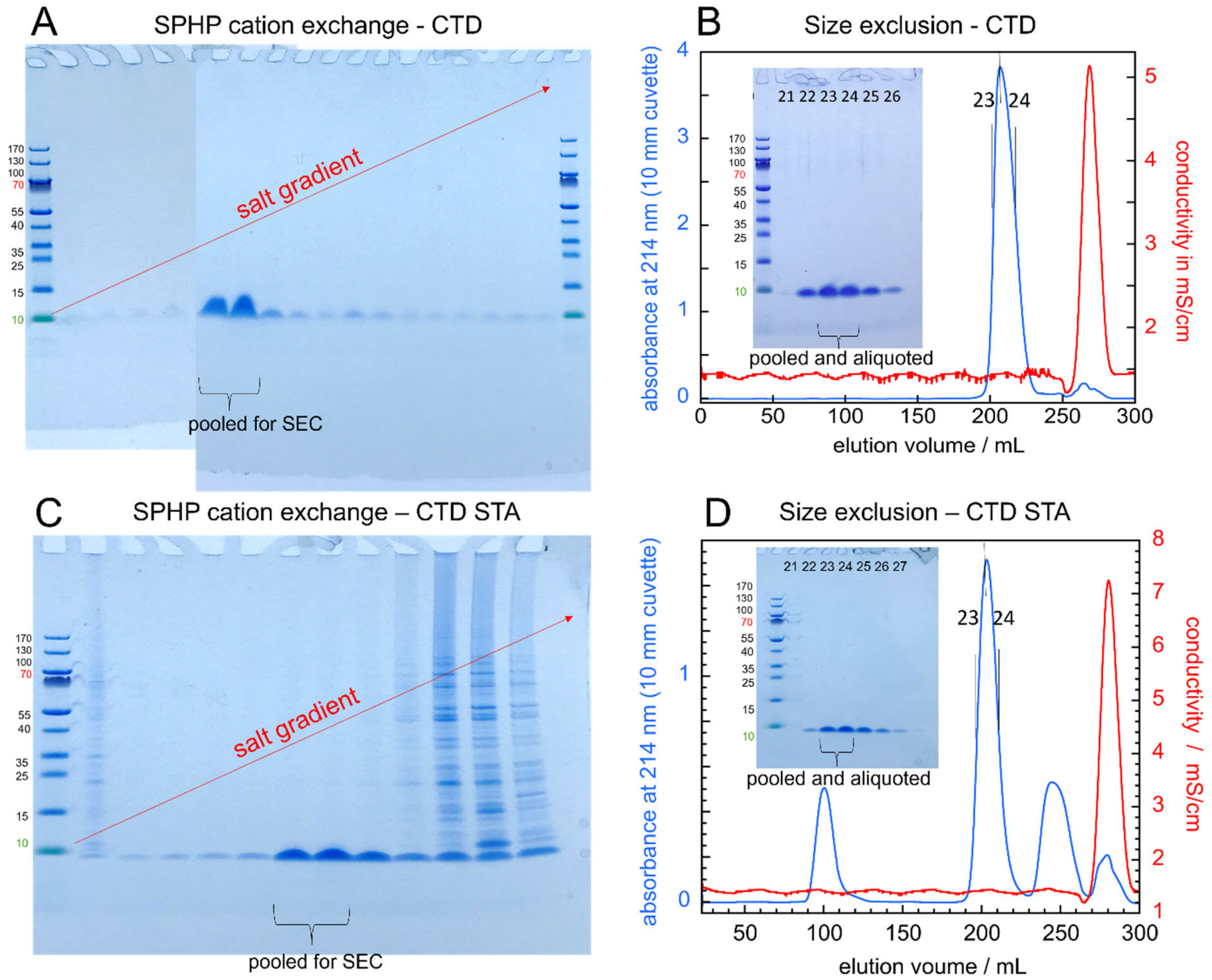

Figure S22. Purification of CTD and CTD STA. Cation exchange of CTD (**A**) and CTD STA (**C**) on a SPHP column. SDS PAGE analysis of fractions from the NaCl gradient elution (0-200 mM NaCl) in in 5 mM Tris/HCl, 1 mM EDTA, pH 8.0. The strongest fractions were pooled, lyophilized, dissolved in 6 M GuHCl and subjected to size exclusion chromatography (SEC) on a Superdex 75 column. The chromatograms for CTD (**B**) and CTD STA (**D**) are displayed as the absorbance at 214 nm and conductivity versus elution volume. The fractions before during and after the protein peak were analyzed by SDS PAGE (insets). The peak fractions, number 23 and 24, were used in the experiments.

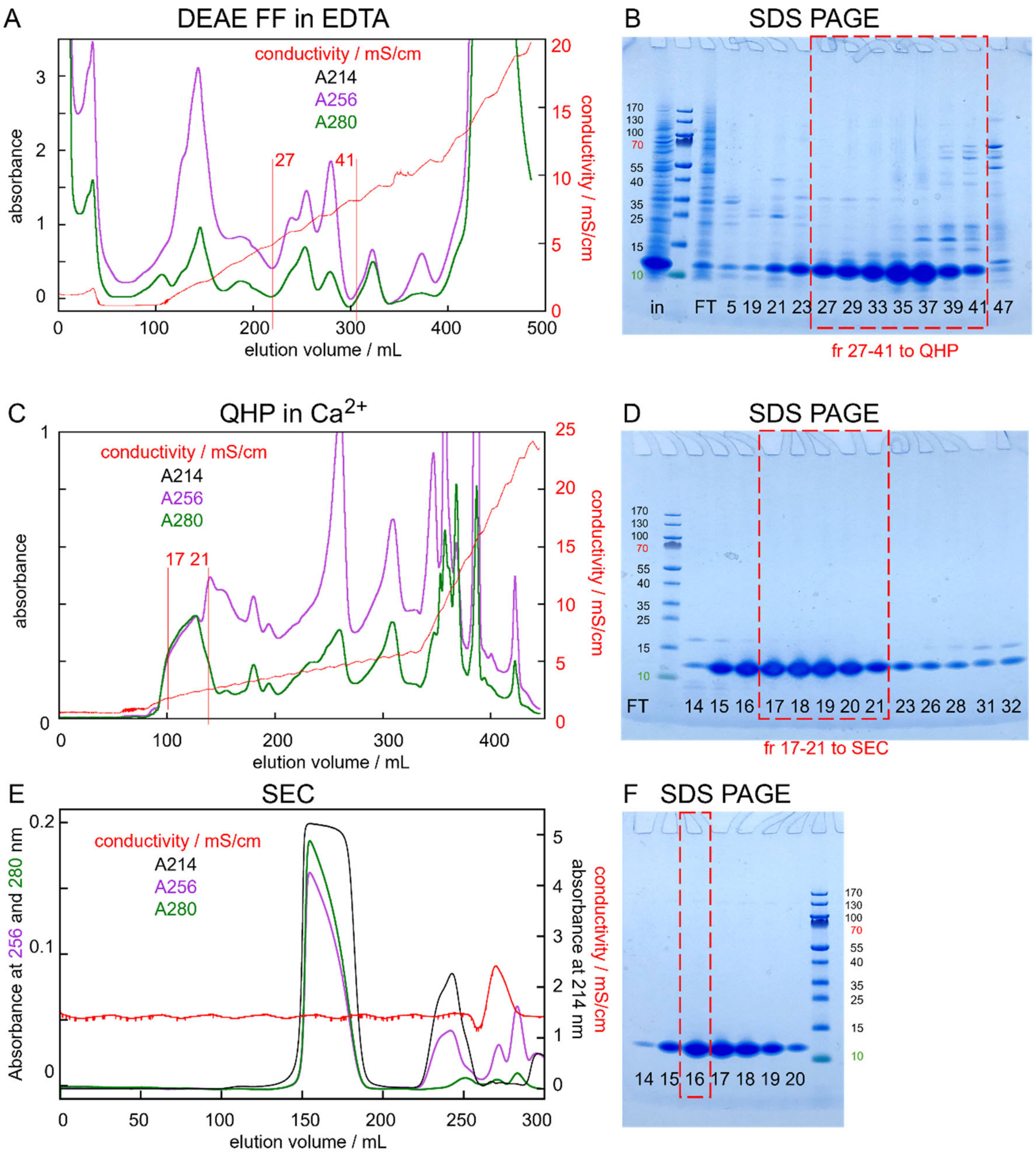

Figure S23. Purification of S100G-CTDβ1-2. A) Chromatogram and **B)** SDS PAGE of selected fractions during salt elution two 20 mL DEAE sepharose FF columns in tandem in 10 mM Tris/HCl, 1 mM EDTA, pH 8.0. Fractions 27-41 were pooled, diluted with H_2O and applied to the QHP column. **C)** Chromatogram and **D)** SDS PAGE of selected fractions during salt elution of a 20 mL Q sepharose HP column in 10 mM Tris/HCl, 1 mM $CaCl_2$, pH 8.0. Fractions 17-21 were pooled, lyophilized and applied to the SEC column. **E)** Chromatogram and **F)** SDS PAGE of selected fractions during elution of a 26 x 600 mm Superdex 75 column in 20 mM sodium phosphate, 0.2 mM EDTA, pH 8.0. Fraction 16 was used in the experiments of the current study.

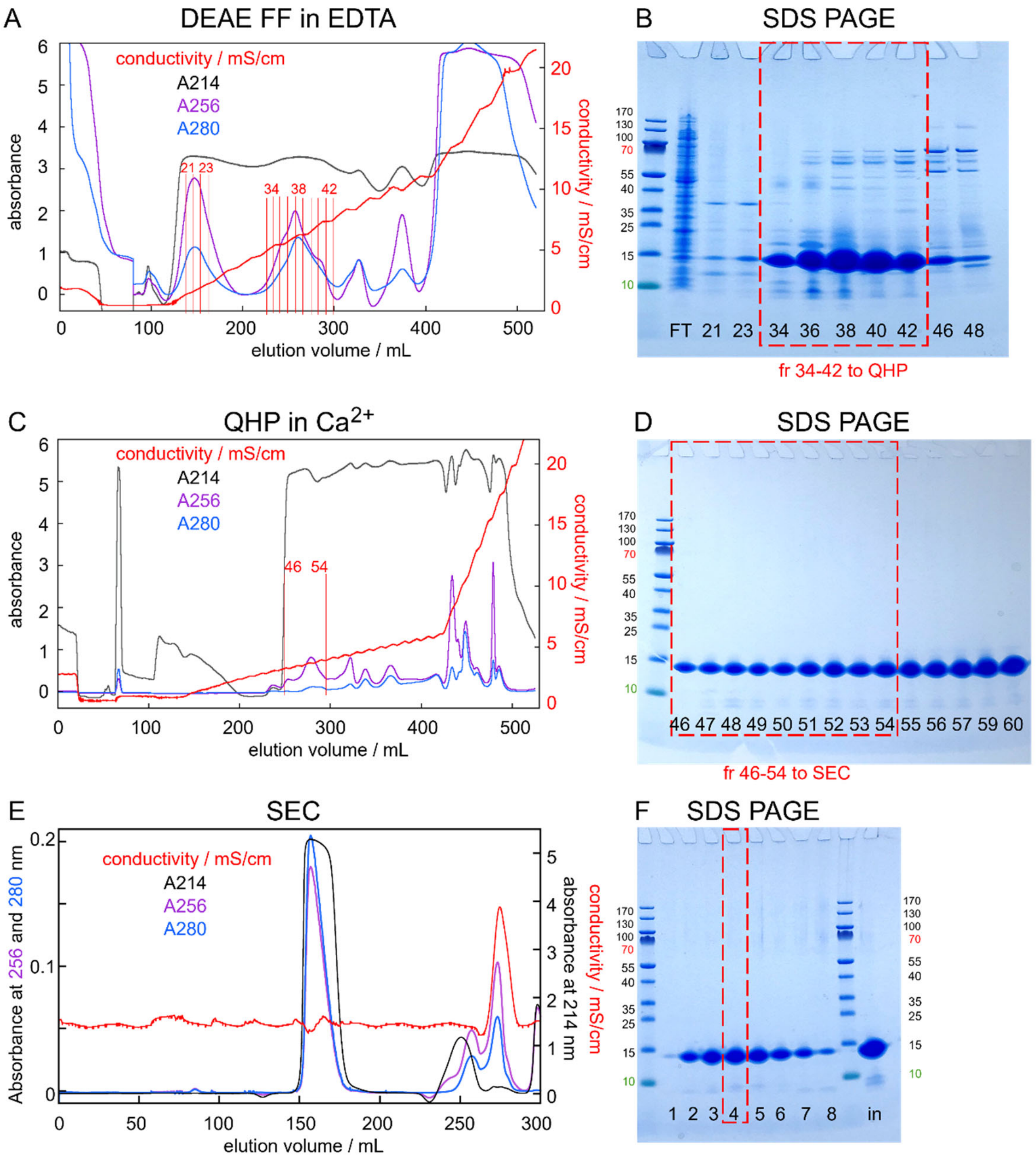

Figure S24. Purification of S100G-CTDβ1-4. A) Chromatogram and **B)** SDS PAGE of selected fractions during salt elution two 20 mL DEAE sepharose FF columns in tandem in 10 mM Tris/HCl, 1 mM EDTA, pH 8.0. Fractions 34-42 were pooled, diluted with H_2O and applied to the QHP column. **C)** Chromatogram and **D)** SDS PAGE of selected fractions during salt elution of a 20 mL Q sepharose HP column in 10 mM Tris/HCl, 1 mM $CaCl_2$, pH 8.0. Fractions 46-54 were pooled, lyophilized and applied to the SEC column. **E)** Chromatogram and **F)** SDS PAGE of selected fractions during elution of a 26 x 600 mm Superdex 75 column in 20 mM sodium phosphate, 0.2 mM EDTA, pH 8.0. Fraction 4 was used in the experiments of the current study.

Supporting Tables (S1-S2)

Table S1. Rate constants from SPR. All values are given with one significant digit.

**Construct**	**kon** / $M^{-1}s^{-1}$	**koff** / s^{-1}	**apparent K** / M^{-1}	**apparent K_D** / M
CTD	400	0.004	$1 \cdot 10^5$	10 µM
CTD STA	500	0.004	$1 \cdot 10^5$	8 µM
S100G-CTDβ1-2	3000	0.006	$2 \cdot 10^5$	2 µM
S100G-CTDβ1-2STA	3000	0.006	$2 \cdot 10^5$	2 µM
S100G-CTDβ1-4	2000	0.006	$3 \cdot 10^5$	3 µM
S100G-CTDβ1-4STA	3000	0.006	$2 \cdot 10^5$	2 µM

Table S2. Crosslinks detected after incubation of Aβ42 with DNAJB6 and CTD, respectively. Approved MS/MS-spectra to validate the crosslinks are supplied in Supporting Figure S10 with requirement for approval of matching >5 fragment ions in each half of the crosslinked peptide. *Lysine residue numbers in CTD refers to the corresponding number in full-length DNAJB6, in Aβ42 refers to peptide without the N-terminal methionine used for recombinant expression. Crosslinks within DNAJB6 and within CTD are not listed.

Detected crosslinks in sample	Precursor mass	Charge	Score	Crosslinked peptides	Lys A*	Lys B*
**Aβ42 and DNAJB6**						
Crosslink Aβ42 to DNAJB6	615.045	4+	36.2	HDSGYEVHHQKL + SISTSTKM	K16	K196
Crosslink Aβ42 to Aβ42	604.608	3+	17.0	MDAEFR + EVHHQKL	N-term	K16
**Aβ42 and CTD**						
Crosslink Aβ42 to CTD	-	-	-	no crosslink detected	-	-
Crosslink Aβ42 to Aβ42	593.525	4+	41.9	MDAEFR + EVHHQKL	N-term	K16

SI References

1. Jumper, J. *et al.* Highly accurate protein structure prediction with AlphaFold. *Nature* **596**, 583–589 (2021).
2. Mirdita, M., Ovchinnikov, S. & Steinegger, M. ColabFold - Making protein folding accessible to all. *bioRxiv* 2021.08.15.456425 (2021) doi:10.1101/2021.08.15.456425.
3. Cohen, S. I. A. *et al.* Proliferation of amyloid-β42 aggregates occurs through a secondary nucleation mechanism. *Proc Natl Acad Sci U S A* **110**, 9758–9763 (2013).
4. Linse, S. Expression and purification of intrinsically disordered Aβ peptide and setup of reproducible aggregation kinetics experiment. *Methods in Molecular Biology* **2141**, 731–754 (2020).
5. Linse, S. *et al.* Electrostatic contributions to the binding of calcium in calbindin D9k. *Biochemistry* **30**, 154–162 (1991).
6. Xue, W. F., Szczepankiewicz, O., C. Bauer, M., Thulin, E. & Linse, S. Intra- versus Intermolecular Interactions in Monellin: Contribution of Surface Charges to Protein Assembly. *J Mol Biol* **358**, 1244–1255 (2006).
7. Ruotolo, B. T., Benesch, J. L. P., Sandercock, A. M., Hyung, S. J. & Robinson, C. v. Ion mobility-mass spectrometry analysis of large protein complexes. *Nat Protoc* **3**, 1139–1152 (2008).
8. Salbo, R. *et al.* Traveling-wave ion mobility mass spectrometry of protein complexes: Accurate calibrated collision cross-sections of human insulin oligomers. *Rapid Communications in Mass Spectrometry* **26**, 1181–1193 (2012).
9. Söderberg, C. A. G. *et al.* Structural modelling of the DNAJB6 oligomeric chaperone shows a peptide-binding cleft lined with conserved S/T-residues at the dimer interface. *Sci Rep* **8**, (2018).
10. Drew, E. D. & Janes, R. W. PDBMD2CD: providing predicted protein circular dichroism spectra from multiple molecular dynamics-generated protein structures. *Nucleic Acids Res* **48**, W17–W24 (2020).
11. Meisl, G. *et al.* Molecular mechanisms of protein aggregation from global fitting of kinetic models. *Nat Protoc* **11**, 252–72 (2016).
12. Linse, S., Thulin, E., Nilsson, H. & Stigler, J. Benefits and constrains of covalency: the role of loop length in protein stability and ligand binding. *Sci Rep* **10**, 20108 (2020).
13. Adair, G. S., Bock, A. v & Field, H. The hemoglobin system: VI. The oxygen dissociation curve of hemoglobin. *Journal of Biological Chemistry* **63**, 529–545 (1925).
14. Österlund, N., Lundqvist, M., Ilag, L. L., Gräslund, A. & Emanuelsson, C. Amyloid-β oligomers are captured by the DNAJB6 chaperone: Direct detection of interactions that can prevent primary nucleation. *Journal of Biological Chemistry* **295**, 8135–8144 (2020).
15. Månsson, C. *et al.* Interaction of the molecular chaperone DNAJB6 with growing amyloid-beta 42 (Aβ42) aggregates leads to sub-stoichiometric inhibition of amyloid formation. *Journal of Biological Chemistry* **289**, 31066–31076 (2014).
16. Månsson, C. *et al.* Conserved S/T Residues of the Human Chaperone DNAJB6 Are Required for Effective Inhibition of Aβ42 Amyloid Fibril Formation. *Biochemistry* **57**, 4891–4892 (2018).
17. Jumper, J. *et al.* Highly accurate protein structure prediction with AlphaFold. *Nature 2021 596:7873* **596**, 583–589 (2021).
18. Marklund, E. G., Degiacomi, M. T., Robinson, C. v., Baldwin, A. J. & Benesch, J. L. P. Collision cross sections for structural proteomics. *Structure* **23**, 791–799 (2015).
19. Nozaki, Y. & Tanford, C. Examination of titration behavior. *Methods Enzymol* **11**, 715–734 (1967).
